# Palladium-bearing intermetallic electride as an efficient and stable catalyst for Suzuki cross-coupling reactions

**DOI:** 10.1038/s41467-019-13679-0

**Published:** 2019-12-11

**Authors:** Tian-Nan Ye, Yangfan Lu, Zewen Xiao, Jiang Li, Takuya Nakao, Hitoshi Abe, Yasuhiro Niwa, Masaaki Kitano, Tomofumi Tada, Hideo Hosono

**Affiliations:** 1 Materials Research Center for Element Strategy, Tokyo Institute of Technology, 4259 Nagatsuta, Midori-ku, Yokohama 226-8503 Japan; 2 Wuhan National Laboratory for Optoelectronics, Huazhong University of Science and Technology, Wuhan, 430074 China; 3 Laboratory for Materials and Structures, Tokyo Institute of Technology, 4259 Nagatsuta, Midori-ku, Yokohama 226-8503 Japan; 4 High Energy Accelerator Research Organization, KEK, 1-1, Oho, Tsukuba, Ibaraki 305-0801 Japan; 5 Department of Materials Structure Science, School of High Energy Accelerator Science, SOKENDAI, The Graduate University for Advanced Studies, 1-1 Oho, Tsukuba, Ibaraki 305-0801 Japan

**Keywords:** Catalyst synthesis, Heterogeneous catalysis, Density functional theory, Chemical engineering

## Abstract

Suzuki cross-coupling reactions catalyzed by palladium are powerful tools for the synthesis of functional organic compounds. Excellent catalytic activity and stability require negatively charged Pd species and the avoidance of metal leaching or clustering in a heterogeneous system. Here we report a Pd-based electride material, Y_3_Pd_2_, in which active Pd atoms are incorporated in a lattice together with Y. As evidenced from detailed characterization and density functional theory (DFT) calculations, Y_3_Pd_2_ realizes negatively charged Pd species, a low work function and a high carrier density, which are expected to be beneficial for the efficient Suzuki coupling reaction of activated aryl halides with various coupling partners under mild conditions. The catalytic activity of Y_3_Pd_2_ is ten times higher than that of pure Pd and the activation energy is lower by nearly 35%. The Y_3_Pd_2_ intermetallic electride catalyst also exhibited extremely good catalytic stability during long-term coupling reactions.

## Abstract (web summary)

In Suzuki coupling reactions, excellent catalytic performance require negatively charged Pd species and the avoidance of metal leaching or clustering. Here the authors implanted Pd sites into an intermetallic electride, Y_3_Pd_2_, which serves as an efficient and stable catalyst for Suzuki coupling reactions.

## Introduction

Palladium-catalyzed Suzuki cross-coupling reactions have drawn considerable attention as a powerful tool for carbon-carbon bond formation, and are extensively used in organic synthesis chemistry^1–4^. To overcome the high activation barriers of the substrates, electron-rich active sites have been proposed in most homogeneous palladium catalysts by the introduction of electron-donating ligands^5^. However, the use of costly and potentially toxic palladium complexes and ligands remains a controversial issue with respect to sustainable chemical industry^6^. In contrast, heterogeneous catalysts are preferred over homogeneous catalysts due to their facile reusability and compatibility with flow systems^7–11^. There have been numerous efforts to develop heterogeneous catalysts for Suzuki cross-coupling reactions, and most of them are based on the design of palladium clusters or nanoparticles with highly electron-donating supports, photogenerated electrons or hot electrons in heterojunctions^9–14^. In particular, Pérez-Ramírez et al. recently developed single-atom catalysts, in which isolated Pd atoms anchored into the exfoliated graphitic carbon nitride, attract considerable attention due to its excellent activity and stability towards Suzuki cross-coupling reaction, surpassing the performance of state-of-the-art homogeneous catalysts and other conventional heterogeneous catalysts^15^. Electron-rich isolated palladium sites with high electron donation ability generally exhibit excellent catalytic activity for Suzuki cross-coupling reactions^16–20^. However, the stability issue for the loaded palladium catalyst particles has not yet been resolved; severe particle aggregation and/or leaching of active Pd sites on the catalyst surface during the reaction leads to poor recyclability^21–23^. There are few approaches to significantly enhance the activity and stability of heterogeneous palladium-based catalysts because the electron density of the active metal is not easily stabilized.

Recently, Nørskov and colleagues reported active and stable cathode catalysts of Pt alloyed with early transition metals, such as lanthanum, scandium, or yttrium for electrocatalytic oxygen reduction reaction^24–27^. When these alloys are used, the binding energy of the oxygen containing species on Pt can be modified moderately by changing the electronic structure of the active Pt sites^28–31^. Our group recently reported that ternary intermetallic compounds (LaCu_0.67_Si_1.33_ and LaCoSi) have a unique activation ability for H_2_ and N_2_ molecules, and thus exhibit excellent catalytic activity for the chemoselective hydrogenation reaction and ammonia synthesis, respectively^32,33^. These intermetallic compounds host solid active sites, high carrier densities, and low work functions (LWFs). The combination of rare earth metal and active transition metal can also induce charge transfer from the rare earth metal to the transition metal, which results in negatively charged active sites. As a consequence, electron transfer from the catalyst to the lowest unoccupied molecular orbitals (LUMOs) of absorbed molecules can be realized, which leads to weakening of the chemical bonds of the absorbed molecules and enables molecular dissociation with lower activation energy (*E*_a_)^34–37^. Inspired by these developments, we have attempted to design a Pd-based intermetallic electride that should have both solid Pd sites and strong electron donation ability associated with a high carrier density and a LWF.

Herein, we propose a Pd-based electride, Y_3_Pd_2_, as an efficient catalyst for Suzuki cross-coupling reactions. The work function of Y_3_Pd_2_ (Φ_WF_ ≈ 3.4 eV) is comparable to that of metallic yttrium (Φ_WF_ ≈ 3.3 eV); however, Y_3_Pd_2_ is stable in air and under the reaction conditions for Suzuki cross-coupling. The measured carrier density is *n*_e_ ≈ 1 × 10^22^ cm^−3^, which is much higher than that for oxide and carbon supports. Together with the negatively charged Pd, associated with different electronegativity between yttrium and palladium, Y_3_Pd_2_ exhibits stable and high catalytic activity for Suzuki cross-coupling reactions. The high carrier density and LWF for Y_3_Pd_2_ enable the activation of aryl halides with a significantly reduced *E*_a_ (about 35% less) compared with that for a pure Pd catalyst, and the turnover frequency (TOF) and recycle number exceed those for other reported Pd-based heterogeneous catalysts under similar reaction conditions. We consider that the electron transfer from the negatively charged Pd to aryl halides could be the key factor for the dissociation of aryl halides and the intrinsic catalytic ability of the active Pd sites involved was significantly enhanced in the Y_3_Pd_2_ intermetallic electride material.

## Results

### The crystal and electronic structures of Y_3_Pd_2_

**Fig. 1:**
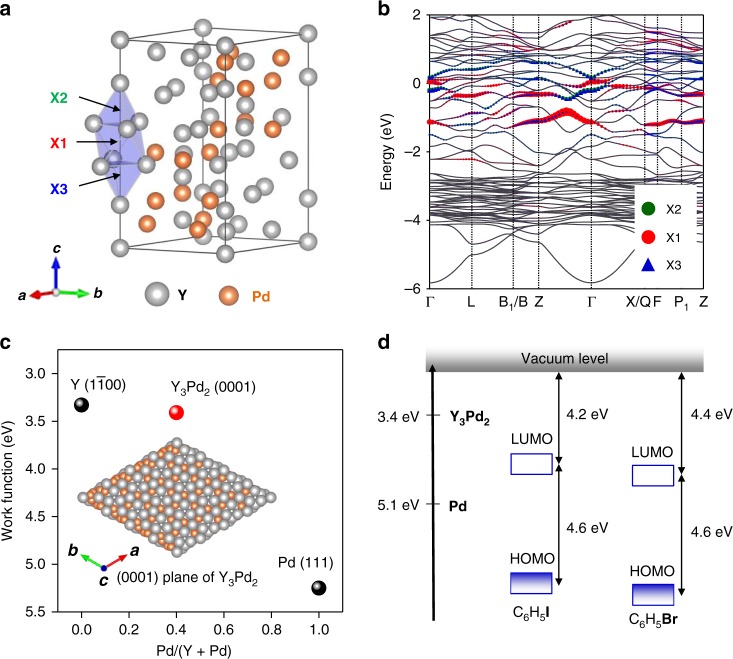
Crystal and electronic structures of Y_3_Pd_2_. **a** Crystal structure of stoichiometric Y_3_Pd_2_. Y and Pd atoms are depicted as gray and orange balls, respectively. X1, X2, and X3 sites are indicated using black solid arrows. **b** Calculated band structure of stoichiometric Y_3_Pd_2_. The contributions of X1, X2, and X3 sites are indicated using red, green and blue dots, respectively. **c** Calculated work functions for the most stable surface, Y_3_Pd_2_(0001). The inset shows the 0001 surface of Y_3_Pd_2_. **d** Comparison of the Fermi level for Y_3_Pd_2_ and the unoccupied (LUMO) states for aryl halides, iodobenzene, and bromobenzene.

Y_3_Pd_2_ crystallizes with a trigonal structure (Fig. 1a), and can be synthesized using arc-melting and subsequent annealing (Supplementary Fig. 1). The obtained powder are ductile and air-water stable. One specific feature of Y_3_Pd_2_ is the presence of interstitial sites. As depicted as X1, X2, and X3 in Fig. 1, yttrium atoms form Y_4_ tetrahedral and Y_6_ octahedral cages. The Y–Y distances for the Y_6_ octahedra and Y_4_ tetrahedra are 3.73 and 3.80 Å, respectively, which is very close to that of the Y_5_Si_3_ electride (3.78 Å)^38^. Consequently, large volumes of Y_4_ tetrahedral and Y_6_ octahedral cages are realized in Y_3_Pd_2_, and thus the X1, X2, and X3 sites can be considered as periodically distributed cavities. Bader charge analysis using pseudo atoms of these interstitial sites revealed that 0.87, 1.05, and 1.05 electrons are confined in the X1, X2, and X3 sites to give a formal valence state of [Y_3_Pd_2_]^+^:e^‒^, which is consistent with the electride concept. Motivated by the presence of interstitial sites, we conducted DFT calculations to clarify the electronic structure. Figure 1b shows that Y_3_Pd_2_ exhibits a metallic band structure. The electronic bands near the Fermi level are partially associated with empty X1, X2, and X3 sites (Supplementary Fig. 2). The chemical environments of the X2 and X3 sites are similar; therefore, these components (green and blue dots) occupy the same positions in the band structure (Supplementary Fig. 3). Partial electron density analysis also shows that the interstitial sites accommodate anionic electrons (Supplementary Fig. 3). These results indicate that the X1, X2, and X3 sites are electrically active, which supports the electride concept in Y_3_Pd_2_. The DFT calculation also showed the electron transfer from yttrium to palladium, pushing down the d-band center of Y_3_Pd_2_ (Supplementary Fig. 2). Thus, by combing with ductile nature, the co-existence of ionic and metallic bond interactions can be expected in Y_3_Pd_2_, which should benefit to realize a solid Pd sites.

As predicted by the DFT calculations, Y_3_Pd_2_ exhibits metallic conductivity, and the carrier density was estimated to be *n*_e_ ≈ 1 × 10^22^ cm^−3^, which is much higher than that for traditional oxides and carbon supports (Supplementary Fig. 4). Furthermore, the work function of Y_3_Pd_2_ (0001), the most stable surface, was calculated to be 3.4 eV (Fig. 1c, Supplementary Fig. 5), which is much smaller than that for pure Pd metal (Φ_WF_ ≈ 5.1 eV, Supplementary Fig. 6), although very close to that for yttrium metal (Φ_WF_ ≈ 3.3 eV, Supplementary Fig. 6). It should be noted that the oxidative addition of aryl halides is the first and the most important step in Suzuki cross-coupling reactions. As typical aryl halides, the estimated LUMO positions of iodobenzene and bromobenzene are around 4.2 eV and 4.4 eV below the vacuum level, respectively, and thus deeper than the Fermi level for Y_3_Pd_2_, but much shallower than that for pure Pd metal (see Fig. 1d). Therefore, the combination of active Pd sites and strong electron donation ability of Y provides surface electrons that are efficiently donated from Y_3_Pd_2_ to the LUMOs of adsorbed aryl halides molecules, which is expected to lower *E*_a_ for the breaking of C−X bonds (X = I and Br).

### Catalytic performance evaluation for Suzuki coupling reactions

**Fig. 2:**
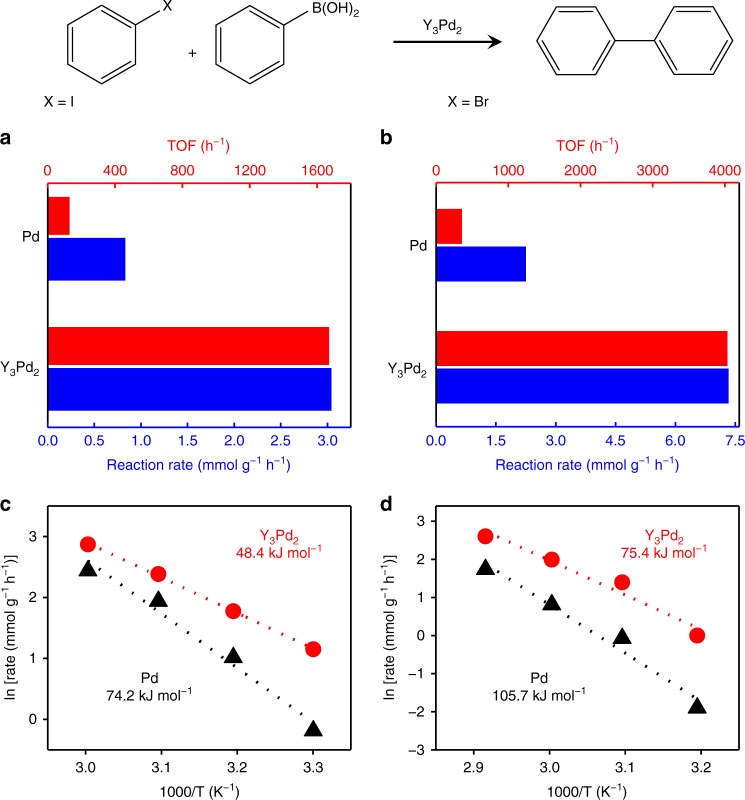
Catalytic performance and kinetic analysis for Suzuki coupling reactions. **a**, **b** Suzuki coupling reaction rates (blue bars) and TOFs (red bars) over Y_3_Pd_2_ and pure Pd metal catalysts. **c**, **d** Apparent *E*_a_s for the Suzuki coupling reaction over Y_3_Pd_2_ and pure Pd metal catalysts. Reaction conditions: Pd (40 mol% relative to organohalide); 0.5 mmol organohalide, 0.8 mmol phenylboronic acid, 1.5 mmol K_2_CO_3_, 5 ml solvent, iodides 30 °C, bromides 60 °C.

The couplings of phenylboronic acid with iodobenzene and bromobenzene are used as model reactions to study the reaction kinetics and catalytic activity. The data in Fig. 2a, b show the Suzuki coupling activity of Y_3_Pd_2_ and pure Pd metal catalysts. For both reactions, the Y_3_Pd_2_ catalyst exhibited rather higher reaction rates than those of pure Pd metals (Supplementary Fig. 7). This can be attributed to the introduced Y species that surrounds the Pd sites, which would significantly improve the catalytic activity. Due to the high quantities of the total Pd amount in the bulk of Y_3_Pd_2_, the turnover frequencies (TOFs) based on total Pd was firstly calculated to be 7.6 h^−1^, which is several times higher than that of pure Pd metal (0.9 h^−1^) with comparable particle size under the same conditions (Table S1, Supplementary Fig. 8). Ullmann homo-coupling of aryl halides can be excluded through the inactive solely iodobenzene coupling reaction and the carbon balance of both catalysts for Suzuki coupling reactions were estimated to be >98% (Supplementary Tables 1, 2). It should be noted that the catalytic reaction process often takes place on the surface of the catalyst and the activity solely depends on the surface exposed active sites. Here, the active ensembles of Pd-Y unit should be regarded as the surface active sites, which can be confirmed by our DFT calculations (see below Supplementary Fig. 14). Therefore, the TOFs were calculated based on each exposed Pd-Y sites. It is evident that the calculated TOFs for Y_3_Pd_2_ are one order of magnitude higher than those for the pure Pd metal catalyst (Supplementary Table 1). Impressively, in terms of TOFs, Y_3_Pd_2_ even outperforms other benchmarked commercial heterogeneous Pd catalysts (Pd/C, Pd/Al_2_O_3_, and Pd-Pb/CaCO_3_) by an order of magnitude (Supplementary Table 3). Such TOF values are also much higher than those of the most reported Pd-based heterogeneous catalysts, including Pd-based photon-assisted catalysts in Suzuki coupling systems under similar reaction conditions (Supplementary Tables 4, 5). Although the highest TOF value was kept by Pd^2+^-exchanged graphite oxide as a heterogeneous catalyst, the deactivation problem still cannot be avoided in heterogeneous system due to the agglomeration of the Pd nanoparticles^9^. However, in Y_3_Pd_2_, the incorporated Pd sites in the crystalline lattice can solve the re-deposition and agglomeration problems in Suzuki coupling reactions which will be discussed below.

While the coupling reaction of chlorobenzene with phenylboronic acid was also tested (Supplementary Table 6), the activity for forming diphenyl was lower than that of bromobenzene and iodobenzene. As widely known in Suzuki coupling reactions, the lower catalytic activity can be attributed to the stronger C−Cl bond of chlorobenzene than that of C−Br and C−I^13^. Thus, breaking C−Cl bond of chlorobenzene needs higher temperature, leading to a low activity between chlorobenzene and phenylboronic acid at mild condition. Here, used Y_3_Pd_2_ is the bulky material, and its specific surface area was much lower than previously studied catalytic system. However, we found the surface area of Y_3_Pd_2_ can be elevated by 8 times through a ball-mill treatment, and accordingly the reaction rate increased in proportion while TOFs remains largely unchanged (Supplementary Table 7 and Supplementary Figs. 9–11). This tendency revealed a very promising potential that the performance may be further improved, e.g., by supporting the material and/or nanocrystallization adjustment of the particle size.

### Kinetic analysis

The kinetics of the Suzuki coupling over Y_3_Pd_2_ and pure Pd metal catalysts were then examined. Based on the organic halide conversion at the start of each reaction, Y_3_Pd_2_ always exhibited higher TOF values than pure Pd metal catalysts (Fig. 2), which implied high activity. Based on the initial reaction rate (*r*_0_) values at different reaction temperatures, a set of Arrhenius plots were produced by plotting ln*r*_0_ as a function of 1/T. The slope of the Arrhenius plot was used to calculate the apparent *E*_a_ for Y_3_Pd_2_ to be 48.4 kJ mol^−1^ for the Suzuki coupling of iodobenzene and phenylboronic acid (Fig. 2c), which was an *E*_a_ reduction of around 35% compared to that for pure Pd metal catalysts (74.2 kJ mol^−1^), which provides evidence that the Suzuki coupling reaction is highly promoted over Y_3_Pd_2_. Similarly, *E*_a_ for the coupling of bromobenzene (Fig. 2d) with phenylboronic acid over Y_3_Pd_2_ follows the same trend, with a reduction of *E*_a_ by about 30% compared with that for pure Pd metal catalysts. Furthermore, the dependence of *r*_0_ on the concentration of aryl halides and phenylboronic acid was examined to determine the kinetic reaction order. As shown in Supplementary Fig. 12, both reactions exhibited a first order relationship with respect to the aryl halide (iodo and bromo) concentration and almost a zero order dependence on the phenylboronic acid concentration. Therefore, the activation of aryl halides (iodo and bromo) acted as the rate controlling step over Y_3_Pd_2_. This is a commonly observed phenomenon for Suzuki cross-coupling reactions over both homogeneous and heterogeneous Pd based catalysts, i.e., the oxidative addition of the aryl halide is the rate-determining step for the catalytic cycle^39–41^. From these kinetic studies, the distinct apparent *E*_a_ values and reaction kinetic orders suggest that the enhanced activity for the Y_3_Pd_2_ catalyst compared to that for pure Pd metal exclusively originates from the promoted activation of the aryl halides. Corresponding DFT simulations was also examined to investigate the activation behaviors of aryl halides on the Y_3_Pd_2_ (0001) surface. The activation process of bromobenzene molecule proceeds with the calculated energy gain of 0.11 eV on Y_3_Pd_2_ (0001), much lower than that of 0.53 eV on Pd (111) (Supplementary Figs. 13, 14). It should be noted that the difference between the calculated and experimental values could be due to the effects which were not taken into account such as solvent effect^42^. Nevertheless, the distinct calculated activation energy difference reasonably agree with the experimental observations. Furthermore, the Bader charge analysis indicates an obvious charge transfer (~0.12 e^−^) from the Y_3_Pd_2_ (0001) surface to the adsorbed aryl halides via the active Pd site (Supplementary Table 8), which is favored for the electron donation to the antibonding orbitals of aryl halides. These results predicted that the activation step of carbon halogen bond on Y_3_Pd_2_ catalyst is remarkably enhanced, explaining the high performance on Suzuki coupling reactions.

Other different Y-Pd binary intermetallic compounds such as Y_3_Pd, Y_3_Pd_4_, and YPd_3_ were also investigated (Supplementary Fig. 15). It is noted that their Pd active sites were also negatively charged with low work function features (Supplementary Figs. 16, 17). We found that all of Y_3_Pd, Y_3_Pd_4_, and YPd_3_ show moderate reaction rates per catalyst weight for Suzuki coupling reaction. Unfortunately, they are much harder to crash than Y_3_Pd_2_, leading to lower surface area and reaction rates. On the other hand, their calculated TOFs are comparable to that of Y_3_Pd_2_, and nearly one order of magnitude higher than pure Pd metal (Supplementary Table 9). Kinetic study also demonstrated that these Y-Pd binary catalysts exhibit the similar activation energies for the Suzuki coupling reactions (Supplementary Fig. 18). These results well suggest the importance of the combination between Y and Pd species in terms of electron density modification, resulting in the significant enhancement of the catalytic activity.

### Spectroscopy characterizations

**Fig. 3:**
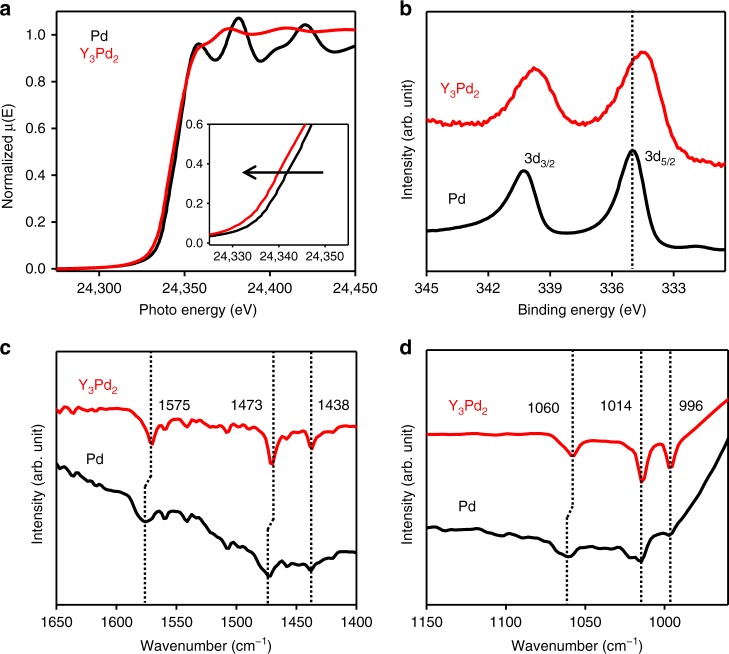
Spectroscopy characterization of Y_3_Pd_2_ and Pd. **a** Pd K-edge XANES spectra and **b** Pd 3*d* XPS spectra for Pd and Y_3_Pd_2_. **c**, **d** DRIFTS spectra for the adsorption of iodobenzene onto Pd and Y_3_Pd_2_ at 25 °C, respectively.

To determine the origin of the superior activation of aryl halides on Y_3_Pd_2_, the electronic structure of the Pd species was further analyzed using X-ray absorption near-edge structure (XANES) and extended X-ray absorption fine structure (EXAFS) spectroscopy. The results indicated that the absorption edge for the Pd species in intermetallic compounds was located at an even lower energy relative to that for the Pd foil, which implies that Pd is negatively charged in Y_3_Pd_2_ (see Fig. 3a). The EXAFS spectrum of the sample was also fitted well by the Y_3_Pd_2_ structure (Supplementary Fig. 19). The intrinsic loss factor *S*_0_^2^ was obtained to be 1.2 showing that the sample consists of bulky Y_3_Pd_2_. This EXAFS fitting result is consistent with the powder X-ray diffraction (XRD) patterns (Supplementary Fig. 1). One might think why long range peaks at around 4 Å are missing. EXAFS FT simulations by FEFF calculations were performed for the ideal Y_3_Pd_2_ structure to check long range interactions^43,44^. The amplitude of each path has a fair value, and the sum of the amplitudes shows a strong peak at ~4 Å (Supplementary Fig. 20). However, when an experimental EXAFS FT is compared with calculated paths, the phases of the paths should be considered. The envelop function of the imaginary parts corresponds to the real EXAFS FT data. The sum of the imaginary parts of the paths and its envelop function are shown in Supplementary Fig. 21. It was revealed that the phases of long range paths are different each other, and the intensities of long range interactions are weakened.

The catalytic process only occurs on the surface of the catalyst; therefore, we also supplied the surface properties as determined by XPS. Figure 3b shows the XPS Pd 3*d* spectra with respect to the binding energy of Y_3_Pd_2_ and pure Pd metal. The obvious shifts of typical Pd 3*d* XPS peaks to lower energy further indicate the elevated electron density of Pd sites, while the Y 3*d* peaks gave a significant shift to higher energy compared to that for Y^0^ 3*d* (Supplementary Fig. 22). Here, it should be noted that the generation of surface yttrium oxides can also be ruled out by the absence of Y–O bonds from the XPS O 1 *s* spectra of Y_3_Pd_2_ (Supplementary Fig. 23). The slightly larger full width at half maximum (FWHM) of Pd 3*d* XPS peaks in Y_3_Pd_2_ compared to pure Pd metal can be ascribed to the co-existence of lowly and fully coordinated Pd species on the surface and in the bulk respectively (Supplementary Fig. 24) which is also confirmed by the Bader charge analysis in Supplementary Table 8, in which the oxidation states of bulk Pd and surface Pd are –1.88 and –1.77, respectively. Bader charge analysis also indicate the oxidation states of the Pd and three different Y atoms (i.e., Y1, Y2, Y3) are –1.88 and +1.19, +1.19, +1.23, respectively, indicating an obviously electron transfer from Y to Pd in the Y_3_Pd_2_ intermetallic (see Supplementary Table 8). These observation combined with the XANES analysis results demonstrate the key role of the rare earth metal (Y) as an electron donor in modifying the electron density and thus the final catalytic activity of the Pd species.

Infrared (IR) spectroscopy has been reported to be a powerful technique for understanding the electronic effects related to adsorbed molecule activation processes on a solid catalyst. Therefore, to elucidate the interaction between the aryl halides and the catalyst, diffuse reflectance infrared Fourier transform spectroscopy (DRIFTS) spectra were measured for iodobenzene adsorbed on Y_3_Pd_2_ and pure Pd metal. The typical C–C stretching in the aromatic vibration bands (νC–C) are in the range of 1400–1600 cm^−1^^45,46^. In the low-frequency region, the main peaks at around 996, 1014, and 1060 cm^−1^ can be assigned to trigonal ring breathing, out-of-plane C–H (γC–H) deformation and halogen-sensitive vibration, respectively^46^. Compared to pure Pd metal, the two frequencies at 1575 and 1473 cm^−1^ involved with the admixture of C–I deformation on Y_3_Pd_2_ exhibited an obvious redshift (see Fig. 3c). Moreover, a clear redshift was also observed for the C–I stretching vibration (1060 cm^−1^) for Y_3_Pd_2_ (see Fig. 3d). Both of these the redshifts imply that C–I bonds in the iodobenzene molecules are weakened by electron donation from Y_3_Pd_2_. Electropositive Y metal generally acts as an electron-donating ligand that increases the electron density of Pd, thereby favoring the back-donation of electrons to the antibonding orbitals of C–I, which accounts for the carbon halogen bond weakening.

### Proposed reaction mechanism

**Fig. 4:**
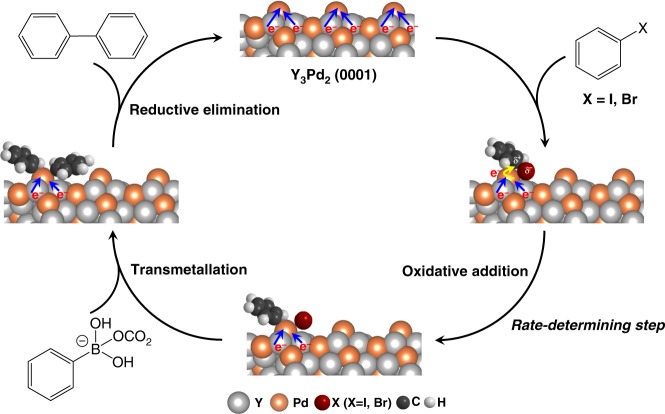
Proposed mechanism. Proposed reaction paths for the Suzuki cross-coupling process over Y_3_Pd_2_.

Figure 4 shows our proposed mechanism for the Suzuki cross-coupling process catalyzed by Y_3_Pd_2_, based on the experimental results. Here, Y_3_Pd_2_ with high electron density of Pd and positively charged Y act as the adsorption sites for the aryl halide (iodobenzene or bromobenzene). Next, the C–X group with an electron withdrawing element X (X = I, Br) of the aryl halide molecules can be preferentially adsorbed on Y^δ+^-Pd^δ−^ sites through strong electrostatic interaction. The negatively charged Pd served as the electron donation sites to weaken the C–X bond, resulting in a significantly suppressed *E*_a_, which is confirmed to be the rate-determining step for the reaction. The subsequent reductive elimination and transmetallation processes are followed by the formation of the final coupling products. Although the detailed mechanism remains undetermined at this stage, it is important to emphasize that electron transfer from the negatively charged Pd to the aryl halide could be the key factor for activation of the aryl halide that results in high catalytic activity for the Suzuki coupling reactions.

### Catalytic Stability

**Fig. 5:**
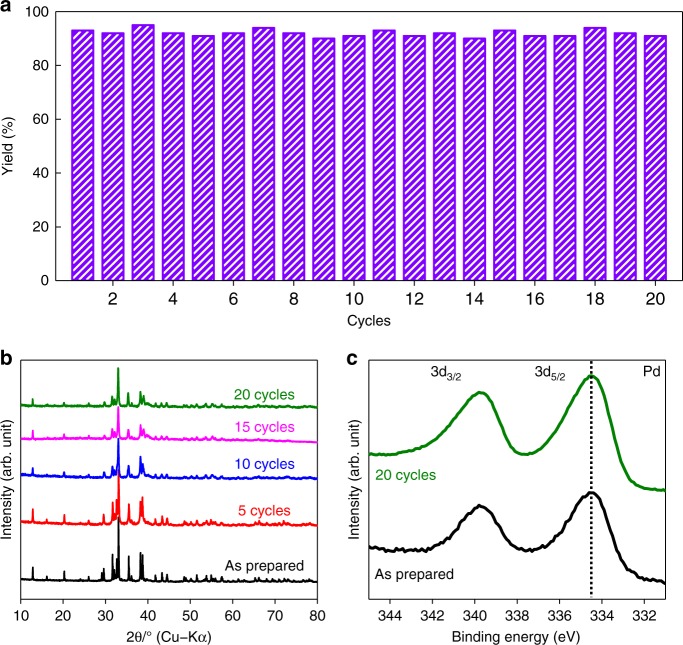
Reusability of Y_3_Pd_2_. **a** Recycling experiment for the Suzuki cross-coupling reaction over Y_3_Pd_2_. Reaction conditions: Pd (40 mol% relative to organohalide); 0.5 mmol iodide, 0.8 mmol phenylboronic acid, 1.5 mmol K_2_CO_3_, 5 ml solvent, 30 °C. **b** Powder XRD patterns for as-prepared and used (after 5, 10, 15, and 20 cycles) Y_3_Pd_2_ catalyst. **c** XPS Pd 3*d* spectra for the as-prepared and used (after 20 cycles) Y_3_Pd_2_ catalyst.

The recyclability of catalysts is one of the most important properties for practical applications. It is worth noting that bare Pd nanostructures or even supported Pd nanoparticles in conventional Suzuki coupling reactions typically suffer from a significant loss in activity because traditional heterogeneous Pd-based catalysts act by the release of small amounts of soluble Pd with the dissolution and re-deposition process, which often induced an aggregation of the loaded Pd species^47–49^. Thus far, recently studied Pd based heterogeneous catalysts could only be reused less than 10 times under mild alkaline conditions^50,51^. In the present work, under the same reaction conditions, Y_3_Pd_2_ could be recycled 20 times without loss of activity, which indicates much higher stability than other reported Pd-based heterogeneous catalysts (see Fig. 5a, Supplementary Tables 4, 5, stability test at low conversion level is also shown in Supplementary Fig. 25). Moreover, significant activity loss was observed on other benchmarked heterogeneous Pd catalysts (Supplementary Fig. 25) and a characteristic Pd particle size change on Pd/C evidenced degradation of the catalyst via metal agglomeration effect (Supplementary Fig. 26), which highlights the obstacle of the sustainability by using heterogeneous catalysts for Suzuki couplings in large scale. In Y_3_Pd_2_, XRD patterns for the used catalyst indicated no obvious change in the crystal structure (Fig. 5b). Most importantly, the XPS results also showed that the Pd species still existed in a negatively charged state, which suggests that the surface electronic structure of Y_3_Pd_2_ is extremely stable (Fig. 5c). Note that the reaction proceeds only in the presence of Y_3_Pd_2_, and no more coupling products can be produced after removal of the catalyst during the reaction process (Supplementary Fig. 27). Inductively coupled plasma (ICP) spectroscopy also revealed that Y and Pd species in the filtrate were below the detection limit (0.007 ppm). Scanning electron microscopy (SEM) observations and energy dispersive X-ray spectroscopy (EDX) analysis also revealed that Y_3_Pd_2_ did not undergo a change in morphology or composition (Supplementary Fig. 28). To shed light on the intrinsic stability of the Y_3_Pd_2_ system, d-band center shift, segregation energy and island formation energy were calculated based on DFT with the well-established methodology^52^. As shown in Supplementary Fig. 29, there is an obvious down-shift of the d-band center (–2.31 eV) for Y_3_Pd_2_ (0001) as compared with Pd (111) (–0.95 eV), indicating a relative lower dissolution potential of the Pd species in Y_3_Pd_2_, which accounts for the high stability during the catalytic reaction^53^. Moreover, the segregation of a Pd atom from the bulk towards the surface requires 1.79‒2.67 eV for Y_3_Pd_2_ (0001) (Supplementary Fig. 30), which is comparable to the values in Pd_3_S system^54^. Also, the formation of Pd islands is also not favored in our case, because the energies required for the exchange of Pd/Y atoms are sufficiently high (0.71‒2.25 eV), as shown in Supplementary Fig. 31. These large segregation and island formation energies are consistent with our initial assessment to Y_3_Pd_2_, in which Pd sites could be stabilized through ionic and metallic bonding within the Y_3_Pd_2_ lattice. These results well explain the excellent stability of the Y_3_Pd_2_ intermetallic electride catalyst.

### Scope of Suzuki coupling reaction

Aromatic and Aliphatic Alcohols. The reaction protocol was further extended to various aryl halides and boronic acids to verify the scope and activity of Suzuki coupling reactions catalyzed by Y_3_Pd_2_ (Table 1). All of the substituted iodobenzenes and bromobenzenes with varied functional groups could be converted to the corresponding coupled products in high yields, regardless of whether electron-donating (CH_3_, OCH_3_, and OH) or electron-withdrawing (F and CF_3_) substituents were present (Supplementary Figs. 32−41). The general applicability of Y_3_Pd_2_ for the coupling of three different types of arylboronic acids bearing electron-donating or electron-withdrawing substituents was also tested (Table 2). It is well known that boronic esters are also applicable to the Suzuki coupling reaction in place of boronic acids because of its high thermal stability and high solubility in organic solvents^55^. Y_3_Pd_2_ catalyst also exhibited a relatively high yield (88%) of biphenyl for the coupling of bromobenzene with phenylboronic acid pinacol ester (Supplementary Table 7 and Supplementary Fig. 42). The calculated TOFs (411.1 h^−1^) were comparable to that of reported Pd single-atom catalyst tested in flow system, while much higher than those of other reported heterogeneous catalysts tested in a batch reactor^15^. The results confirmed the general applicability of the Y_3_Pd_2_ catalyst with high chemoselectivity and functional group tolerance.

**Table 1:**
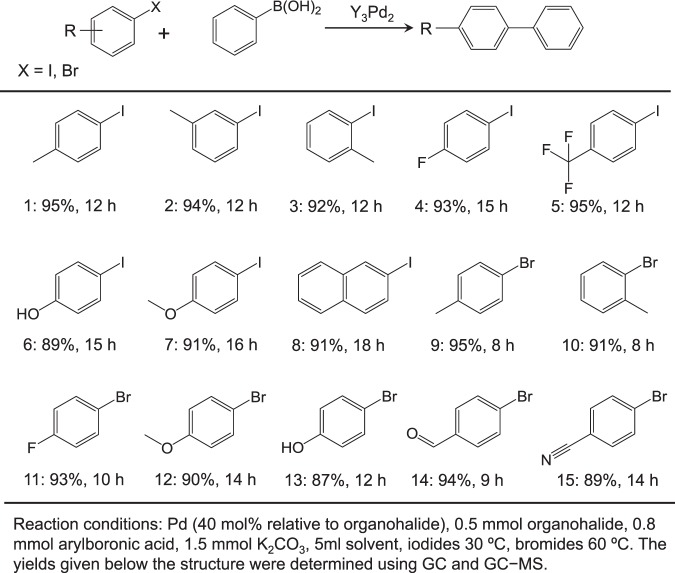
Suzuki cross-coupling reactions for various aryl halides over Y_3_Pd_2_.

**Table 2:**
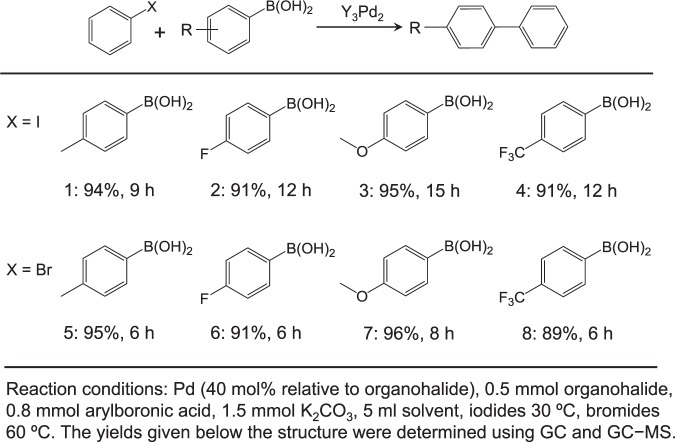
Suzuki cross-coupling reactions of various arylboronic acids with aryl halides over Y_3_Pd_2_.

## Discussion

Guided by the active sites integrated in the intermetallic electride concept, an intermetallic electride catalyst, Y_3_Pd_2_, was developed for Suzuki cross-coupling. The in situ stable intermetallic electride Y_3_Pd_2_ possesses both high carrier density and a LWF, and thus exhibit much higher catalytic activity than Pd metals and other conventional Pd based supported catalysts. Mechanistic studies demonstrated that negatively charged Pd sites induce electron injection into the antibonding orbitals of C–X, significantly promoting the activation of aryl halides, which is the rate-determining step for the investigated Suzuki cross-coupling reactions. Given the heterogeneous nature of the catalyst, Y_3_Pd_2_ could be easily separated and recycled for more than twenty cycles without significant loss in activity. Our results show the possibility of tailoring the electronic structure of active transition metals such as Pd by the formation of intermetallic compounds with early transition elements—an approach that should be broadly applicable for the tuning of catalytic properties.

## Methods

### Sample preparation

Elemental yttrium (ingot) and palladium (ingot and powder) used in this study were purchased from Kojundo Chemical Lab. Co., Ltd. (Japan), and directly used without any pretreatments. Y_3_Pd_2_ was directly synthesized by arc-melting yttrium and palladium ingots with the ratio of 3.1: 2 under an argon atmosphere. The ingot obtained was grounded using an agate motor in Ar filled glove box. The obtained powder was then pelletized and sealed into an argon filled stainless tube, and annealed at 900 °C for 90 h. Stainless tubes were sintered in nitrogen atmosphere in order to prevent their oxidation. Y_3_Pd, Y_3_Pd_4_, and YPd_3_ were prepared by arc-melting method similar with that for the preparation of Y_3_Pd_2_. Ball-milling of Y_3_Pd_2_ was conducted using heptane and ethanol as solvents under argon atmosphere, and the rotation rate was set to 150 rpm. In order to avoid overheating, Y_3_Pd_2_ was milled for 10 min with 10 min of interval, and this sequence was 10 times repeated. Pd/C, Pd/Al_2_O_3_, and Pd–Pb/CaCO_3_ used in this study were purchased from Sigma-Aldrich, and directly used without any pretreatments.

### Physical property measurements

The electronic transport properties, magnetic susceptibility and heat capacity were measured using a physical property measurement system (PPMS) and a superconducting quantum interference device (SQUID) vibrating sample magnetometer (SVSM) from Quantum Design.

### General procedure for Suzuki cross-coupling reactions

All reactions were conducted in a 25 ml stainless steel autoclave fitted with a glass mantel and a magnetic stirrer. In a typical reaction, 0.5 mmol organohalide, 0.8 mmol phenylboronic acid, 1.5 mmol K_2_CO_3_, and Pd (40 mol% relative to organohalide) catalyst were mixed in 5 ml of solvent. The autoclave was then flushed three times with Ar and reactions were performed in the autoclave at about 30–70 °C. The products were analyzed using gas chromatography (GC) and product identification was further confirmed from gas chromatography-mass spectrometry (GC-MS) measurements. The TOF was calculated from the reaction rate at a low conversion level derived from the number of Pd atoms exposed to the catalyst surface (Eqs. 1 and 2):

1 $${\mathrm{TOF}}\,=\,n_0C/tn_{{\mathrm{catalyst}}},$$

2 $$n_{{\mathrm{cat}}}\,=\,m_{{\mathrm{cat}}}N_{{\mathrm{Pd}}\,{\mathrm{sites}}}/N_{\mathrm{A}},$$

where *n*_0_ is the initial number of moles of substrate, *C* is the conversion of the substrate at reaction time *t*, *n*_cat_ is the number of moles of Pd atoms exposed on the surface, *m*_cat_ is the weight of the catalyst, *N*_Pd sites_ is the amount of exposed Pd atoms per gram of catalyst, and *N*_A_ is Avogadro’s constant.

The number of moles of Pd atoms exposed on the surface was estimated by experimental measurement and theoretical prediction, respectively. For experimental measurement, the CO-pulse chemisorption (BELCAT-A, BEL, Japan) was measured at 50 °C using a He flow of 30 ml min^−1^ and pulses of 0.36 ml (9.88% CO in He). Prior to the analysis, the catalyst was treated with flowing Ar (50 ml min^−1^) at 400 °C for 30 min and then flowing He (50 ml min^−1^) at 400 °C for 15 min. To calculate the metal dispersion, a stoichiometry of Pd/CO = 1 was assumed. Because each Pd site was isolated by the surrounded Y atoms in the most stable surface Y_3_Pd_2_ (0001). The nearest separation between surface isolated Pd sites is ~0.43 nm, sufficiently longer than the bond length of CO molecule (~0.11 nm). In addition, the CO adsorption to Y powder was below the detection limit at the same condition. Therefore, it is reasonably assume each active ensemble of Pd-Y corresponds to one CO molecule. For theoretical prediction, both of the Pd atom numbers in one unit cell of Y_3_Pd_2_ crystal and surface area of the corresponding unit cell were calculated. Then the number of the Pd atoms per unit cell’s surface area were extended to the measured specific surface area of the experimentally prepared Y_3_Pd_2_ powder.

For the recycling study, the Suzuki coupling reaction was performed with organohalide and phenylboronic acid, maintaining the same reaction conditions as described above except using recycled catalyst. After each completion of the reaction, the catalyst powder was washed by ethanol with a certain amount of water several times to remove the organic and inorganic impurities from the used catalyst. Finally, the resultant powder was dried in a vacuum at room temperature, weighed, and reused in the next run.

### Sample characterization

The crystal structure was analyzed using an X-ray powder diffractometer (D8 ADVANCE, Bruker) with monochromated Cu Kα radiation (*λ* = 0.15418 nm). Nitrogen sorption measurements (BELSORP-mini II, BEL) were performed to evaluate the Brunauer–Emmett–Teller surface area of the catalysts. The surface morphology of the catalyst samples was investigated using high-angle annular dark field scanning transmission electron microscopy (HAADF-STEM; JEM-2010F, Jeol). XPS (ESCA-3200, Shimadzu) measurements were performed using Mg Kα radiation at <10^−6^ Pa (8 kV bias voltage applied to the X-ray source). Nuclear magnetic resonance (NMR) spectra were recorded on a Bruker Ultrashield Plus 400 instrument (Bruker Corporation) using tetramethylsilane as an internal standard (400 and 100 MHz for ^1^H NMR and ^13^C NMR, respectively). X-ray absorption fine-structure (XAFS) measurements were performed on the AR-NW10A beamline of the Photon Factory Advanced Ring at the Institute of Materials Structure Science, High Energy Accelerator Research Organization, Tsukuba, Japan. A Si (311) double-crystal monochromator was used to obtain a monochromated X-ray beam, and spectra were obtained in transmission mode. Y_3_Pd_2_ with BN (dried at 300 °C) were mixed in an argon-filled glovebox and the mixture was pressed with a hand press apparatus to obtain a pellet sample, which was sealed in a plastic bag for measurement. XAFS spectra were analyzed using the Athena and Artemis software packages^43^. The FEFF6 code^44^ was used to calculate the theoretical spectra.

### DFT calculations

DFT calculations were performed using the generalized gradient approximation (GGA) with the Perdew−Burke−Ernzerhof (PBE)^56^ functional and the projector augmented plane-wave method implemented in the VASP 5.4 code^57^. The plane-wave cutoff energy was set to 500 eV. Γ-centered k-meshes with a k-spacing of 0.1 Å^–1^ were employed to sample the Brillouin zones. The bulk structures were relaxed until the total force on each atom was less than 0.01 eV Å^–1^. A Bader charge analysis was conducted using the Bader program^58^. Calculations for slab supercell models, which consist of atomic layers with thicknesses of around 10 Å and a vacuum layer with a thickness of 15 Å, were performed to determine the work functions and formation energies for different surfaces. The energy barriers of the activation of aryl halides were calculated using the nudged elastic band method implemented in the Vienna Ab initio Simulation Package with 10 replicas, which included initial and final structures and 8 nudged intermediate images.

### FT-IR measurements

DRIFT spectra were measured using a spectrometer (FT/IR-6100, Jasco) equipped with a mercury–cadmium–tellurium detector at a resolution of 4 cm^−1^. An alumina sample cup containing approximately 30 mg of catalyst was introduced into a water-cooled stainless steel heat chamber equipped with KBr windows (STJ-0123-HP-LTV, S.T. Japan). The sample was heated at 200 °C under vacuum for 2 h and then cooled to room temperature. After the pretreatment, iodobenzene was supplied into the system. The infrared spectrum of the sample at room temperature prior to the adsorption of substrates was used as the background for the difference spectra obtained by subtraction of the background from the spectra of the catalyst samples with adsorbed substrate.

## Supplementary information

Supplementary Information

## Data Availability

The data that support the findings of this study are available from the corresponding authors on reasonable request.

## References

[1] Miyaura N, Suzuki A (1995). Palladium-catalyzed cross-coupling reactions of organoboron compounds. Chem. Rev..

[2] Fihri A, Bouhrara M, Nekoueishahraki B, Basset J-M, Polshettiwar V (2011). Nanocatalysts for Suzuki cross-coupling reactions. Chem. Soc. Rev..

[3] Molnár Á (2011). Efficient, selective, and recyclable palladium catalysts in carboncarbon coupling reactions. Chem. Rev..

[4] Glasspoole BW, Crudden CM (2011). The final frontier. Nat. Chem..

[5] Buchwald SL (2008). Cross coupling. Acc. Chem. Res..

[6] Yin LX, Liebscher J (2007). Carbon−carbon coupling reactions catalyzed by heterogeneous palladium catalysts. Chem. Rev..

[7] Grirrane A, Corma A, Garcıá H (2008). Gold-catalyzed synthesis of aromatic azo compounds from anilines and nitroaromatics. Science.

[8] Kesavan L (2011). Solvent-free oxidation of primary carbon-hydrogen bonds in toluene using Au-Pd alloy nanoparticles. Science.

[9] Scheuermann GM, Rumi L, Steurer P, Bannwarth W, Mulhaupt R (2009). Palladium nanoparticles on graphite oxide and its functionalized graphene derivatives as highly active catalysts for the Suzuki2Miyaura coupling reaction. J. Am. Chem. Soc..

[10] Noël T, Kuhn S, Musacchio AJ, Jensen KF, Buchwald SL (2011). Suzuki-miyaura cross-coupling reactions in flow: multistep synthesis enabled by a microfluidic extraction. Angew. Chem. Int. Ed..

[11] Yuan B, Pan Y, Li Y, Yin B, Jiang H (2010). A highly active heterogeneous palladium catalyst for the suzuki–miyaura and ullmann coupling reactions of aryl chlorides in aqueous media. Angew. Chem. Int. Ed..

[12] Sarina S (2013). Enhancing catalytic performance of palladium in gold and palladium alloy nanoparticles for organic synthesis reactions through visible light irradiation at ambient temperatures. J. Am. Chem. Soc..

[13] Leyva-Pérez A, Oliver-Meseguer J, Rubio-Marqués P, Corma A (2013). Water-stabilized three- and four- atom palladium clusters as highly active catalytic species in ligand-free C-C cross-coupling reactions. Angew. Chem. Int. Ed..

[14] Wang F (2014). A polyphenylene support for Pd catalysts with exceptional catalytic activity. Angew. Chem. Int. Ed..

[15] Chen Z (2018). A heterogeneous single-atom palladium catalyst surpassing homogeneous systems for Suzuki coupling. Nat. Nanotechnol..

[16] Fu GC (2008). The development of versatile methods for palladium-catalyzed coupling reactions of aryl electrophiles through the use of P(t-Bu)3 and PCy3 as Ligands. ACC Chem. Res..

[17] Mitchell S, Vorobyeva E, Pérez-Ramírez J (2018). The multifaceted reactivity of single-atom heterogeneous catalysts. Angew. Chem. Int. Ed..

[18] Li XH, Baar M, Blechert S, Antonietti M (2013). Facilitating room-temperature Suzuki coupling reaction with light: Mott-Schottky photocatalyst for C-C-coupling. Sci. Rep..

[19] Raza F (2017). Structuring Pd nanoparticles on 2H-WS2 nanosheets induces excellent photocatalytic activity for cross-coupling reactions under visible light. J. Am. Chem. Soc..

[20] Guo J (2017). Boosting hot electrons in hetero-superstructures for plasmon-enhanced catalysis. J. Am. Chem. Soc..

[21] MacQuarrie S (2008). Visual observation of redistribution and dissolution of palladium during the suzuki–miyaura reaction. Angew. Chem. Int. Ed..

[22] Shao LD (2011). Nanosizing intermetallic compounds onto carbon nanotubes: active and aelective hydrogenation catalysts. Angew. Chem. Int. Ed..

[23] Wan Y (2009). Ordered mesoporous Pd/silica−carbon as a highly active heterogeneous catalyst for coupling reaction of chlorobenzene in aqueous media. J. Am. Chem. Soc..

[24] Greeley J (2009). Alloys of platinum and early transition metals as oxygen reduction electrocatalysts. Nat. Chem..

[25] Escudero-Escribano M (2016). Tuning the activity of Pt alloy electrocatalysts by means of the lanthanide contraction. Science.

[26] Stephens IEL, Bondarenko AS, Grønbjerg U, Rossmeisl J, Chorkendorff I (2012). Understanding the electrocatalysis of oxygen reduction on platinum and its alloys. Energy Environ. Sci..

[27] Stamenkovic VR (2007). Improved oxygen reduction activity on Pt3Ni(111) via increased surface site availability. Science.

[28] Zhang J, Sasaki K, Sutter E, Adzic RR (2007). Stabilization of platinum oxygen-reduction electrocatalysts using gold clusters. Science.

[29] Hernandez-Fernandez P (2014). Mass-selected nanoparticles of PtxY as model catalysts for oxygen electroreduction. Nat. Chem..

[30] Wang SJ (2012). Role of electronic perturbation in stability and activity of Pt-based alloy nanocatalysts for oxygen reduction. J. Am. Chem. Soc..

[31] Roy C (2018). Scalable synthesis of carbon-supported platinum–lanthanide and− rare-earth alloys for oxygen reduction. ACS Catal..

[32] Gong Y (2018). Ternary intermetallic LaCoSi as a catalyst for N2 activation. Nat. Catal..

[33] Ye TN (2017). Copper-based intermetallic electride catalyst for chemoselective hydrogenation reactions. J. Am. Chem. Soc..

[34] Kitano M (2012). Ammonia synthesis using a stable electride as an electron donor and reversible hydrogen store. Nat. Chem..

[35] Kitano M (2015). Electride support boosts nitrogen dissociation over ruthenium catalyst and shifts the bottleneck in ammonia synthesis. Nat. Commun..

[36] Ye TN, Li J, Kitano M, Sasase M, Hosono H (2016). Electronic interactions between a stable electride and a nano-alloy control the chemoselective reduction reaction. Chem. Sci..

[37] Ye TN, Li J, Kitano M, Hosono H (2017). Unique nanocages of 12CaO·7Al2O3 boost heterolytic hydrogen activation and selective hydrogenation of heteroarenes over ruthenium catalyst. Green. Chem..

[38] Lu YF (2016). Water durable electride Y5Si3: electronic structure and catalytic activity for ammonia synthesis. J. Am. Chem. Soc..

[39] Wang F (2013). Plasmonic harvesting of light energy for Suzuki coupling reactions. J. Am. Chem. Soc..

[40] Zhang X (2018). C–C coupling on single-atom-based heterogeneous catalyst. J. Am. Chem. Soc..

[41] Xiao Q (2014). Visible light-driven cross-coupling reactions at lower temperatures using a photocatalyst of palladium and gold alloy nanoparticles. ACS Catal..

[42] Proutiere F, Schoenebeck F (2011). Solvent effect on palladium-catalyzed cross-coupling reactions and implications on the active catalytic species. Angew. Chem. Int. Ed..

[43] Ravel B, Newville M (2005). ATHENA, ARTEMIS, HEPHAESTUS: data analysis for X-ray absorption spectroscopy using IFEFFIT. J. Synchrotron Radiat..

[44] Zabinsky SI, Rehr JJ, Ankudinov A, Albers RC, Eller MJ (1995). Multiple-scattering calculations of x-ray-absorption spectra. Phys. Rev. B.

[45] Wu W, Liao L, Lien C, Lin J (2001). FTIR study of adsorption, thermal reactions and photochemistry of benzene on powdered TiO2. Phys. Chem. Chem. Phys..

[46] Whiffen, D. H. Vibrational frequencies and thermodynamic properties of fluoro-, chloro-, bromo-, and iodo-benzene. *J. Chem. Soc. ***273**, 1350−1356 (1956).

[47] Kohler K, Kleist W, Prockl SS (2007). Genesis of coordinatively unsaturated palladium complexes dissolved from solid precursors during heck coupling reactions and their role as catalytically active species. Inorg. Chem..

[48] Zhao FY, Shirai M, Ikushima Y, Arai M (2002). The leaching and re-deposition of metal species from and onto conventional supported palladium catalysts in the Heck reaction of iodobenzene and methyl acrylate in N-methylpyrrolidone. J. Mol. Catal. A.

[49] Davies IW, Matty L, Hughes DL, Reider PJ (2001). Are heterogeneous catalysts precursors to homogeneous catalysts?. J. Am. Chem. Soc..

[50] Soomro SS, Ansari FL, Chatziapostolou K, Köhler K (2010). Palladium leaching dependent on reaction parameters in Suzuki–Miyaura coupling reactions catalyzed by palladium supported on alumina under mild reaction conditions. J. Catal..

[51] Siamaki AR, Khder AERS, Abdelsayed V, El-Shall MS, Gupton BF (2011). Microwave-assisted synthesis of palladium nanoparticles supported on graphene: a highly active and recyclable catalyst for carbon–carbon cross-coupling reactions. J. Catal..

[52] López N, Vargas-Fuentes C (2012). Promoters in the hydrogenation of alkynes in mixtures: insights from density functional theory. Chem. Commun..

[53] Jinnouchi R, Toyoda E, Hatanaka T, Morimoto Y (2010). First principles calculations on site-dependent dissolution potentials of supported and unsupported Pt particles. J. Phys. Chem. C..

[54] Albani D (2018). Selective ensembles in supported palladium sulfide nanoparticles for alkyne semi-hydrogenation. Nat. Commun..

[55] Kitamura Y (2007). Heterogeneous Pd/C-catalyzed ligand-free Suzuki-Miyaura coupling reaction using aryl boronic esters. Tetrahedron.

[56] Perdew JP, Burke K, Ernzerhof M (1996). Generalized gradient approximation made simple. Phys. Rev. Lett..

[57] Kresse G, Furthmüller J (1996). Efficient iterative schemes for ab initio total-energy calculations using a plane-wave basis set. Phys. Rev. B.

[58] Tang W, Sanville E, Henkelman G (2009). A grid-based Bader analysis algorithm without lattice bias. J. Phys. Condens. Matter.

