# Supplementary material for: Palladium-bearing intermetallic electride as an efficient and stable catalyst for Suzuki cross-coupling reactions
Source: Nat Commun. 2019 Dec 11;10:5653. doi: 10.1038/s41467-019-13679-0 (PMC6906439; doi:10.1038/s41467-019-13679-0)
Supplement: Supplementary file 1 — Supplementary Information [file 41467_2019_13679_MOESM1_ESM.pdf]

Supplementary Information

Palladium-bearing intermetallic electride as efficient and stable catalyst for Suzuki cross-coupling reactions

Ye et al.

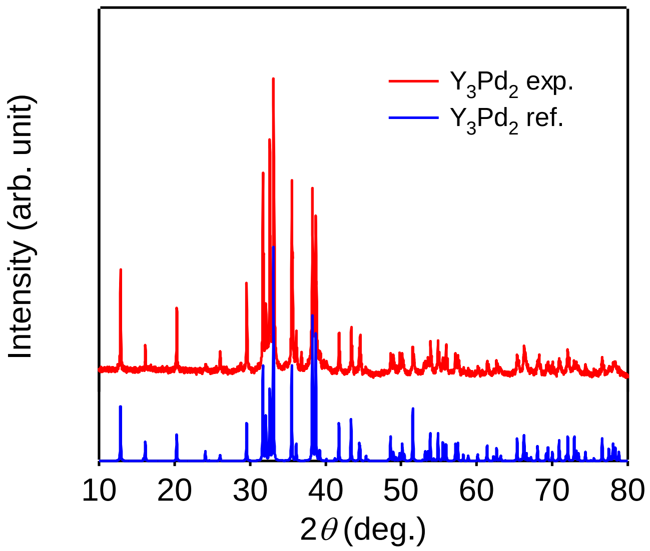

Supplementary Figure 1 | Powder XRD pattern of Y_3Pd_2. The powder XRD pattern of the as-prepared Y_3Pd_2. The red and blue solid lines represent experimentally obtained and calculated pattern.

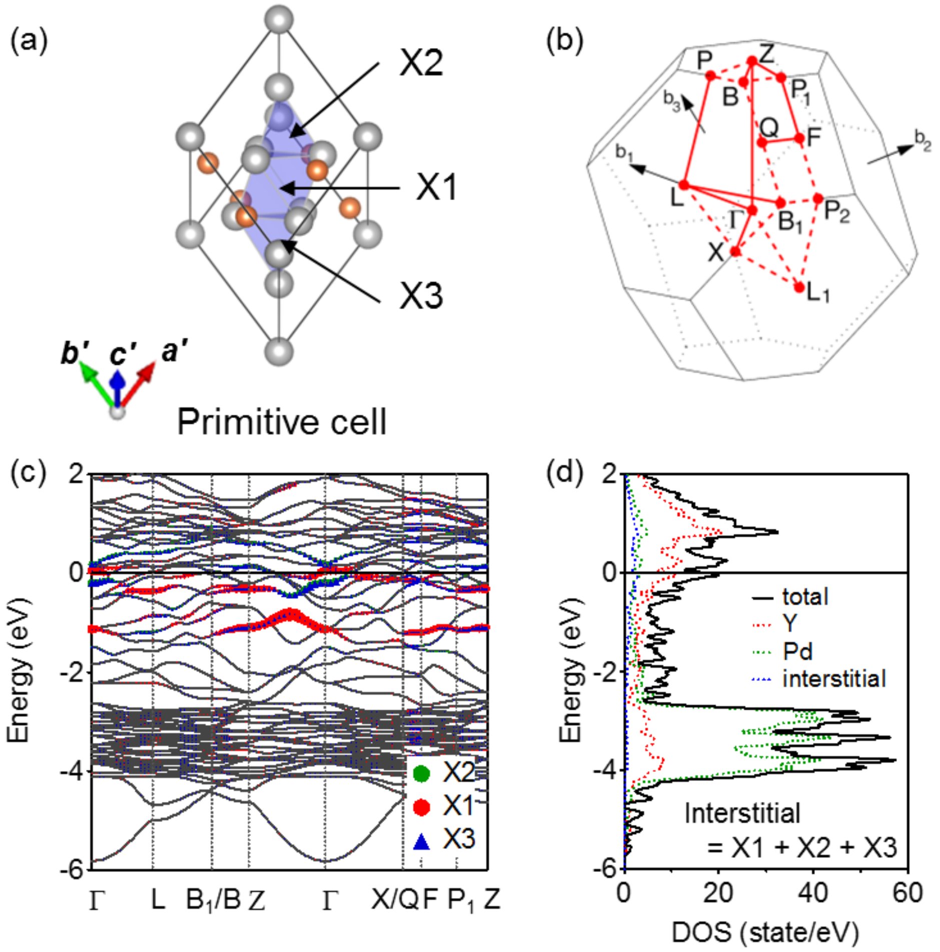

Supplementary Figure 2 | Calculated electronic structure of Y_3Pd_2. (a) Crystal structure (Primitive cell) of Y_3Pd_2 and (b) The geometry of the first Brillouin zones of Y_3Pd_2 based on the primitive cell. (c) Electronic band structure, and (c) DOSs for the stoichiometric Y_3Pd_2. The contribution of X1, X2 and X3 sites are emphasized using red, green and blue dots in (c).

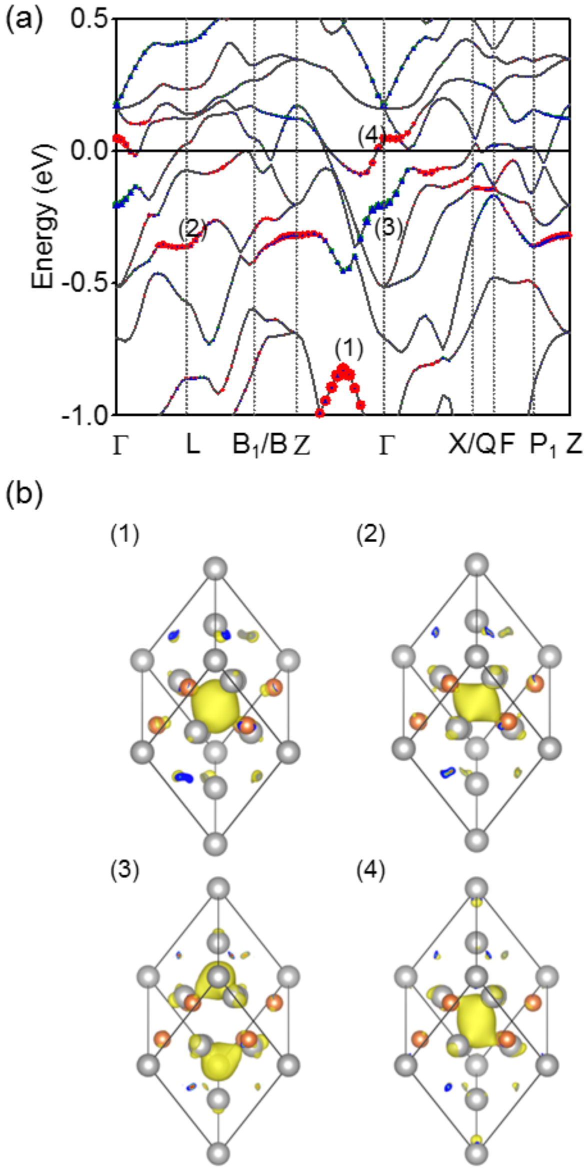

Supplementary Figure 3 (a) The calculated electronic structure of Y_3Pd_2 with narrower energy range. The contribution of X1, X2 and X3 sites are emphasized using red, green and blue dots. (b) Real space electron distribution at corresponding energy bands.

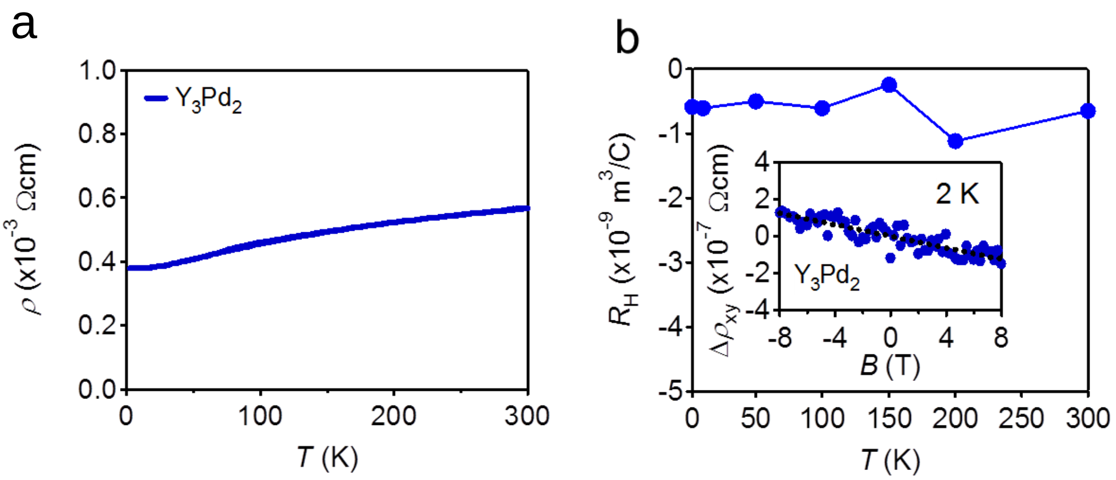

Supplementary Figure 4 | Physical properties of Y_3Pd_2. a, Electrical resistivity and b, Hall coefficient of Y3Pd2. The inset of represents Hall resistivity measured at 2 K.

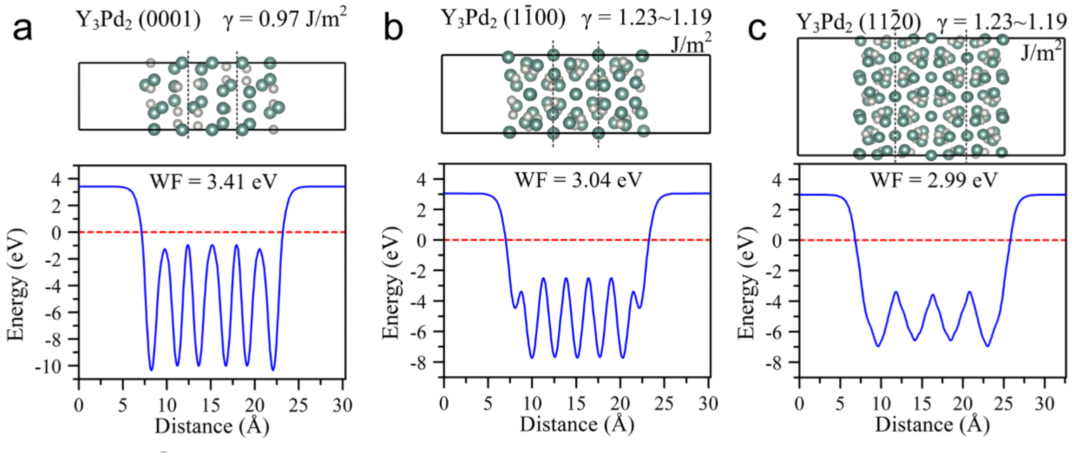

Supplementary Figure 5 | Calculated work functions of Y_3Pd_2. The corresponding slab models and surface energies are also given. The red dashed lines indicate the Fermi levels.

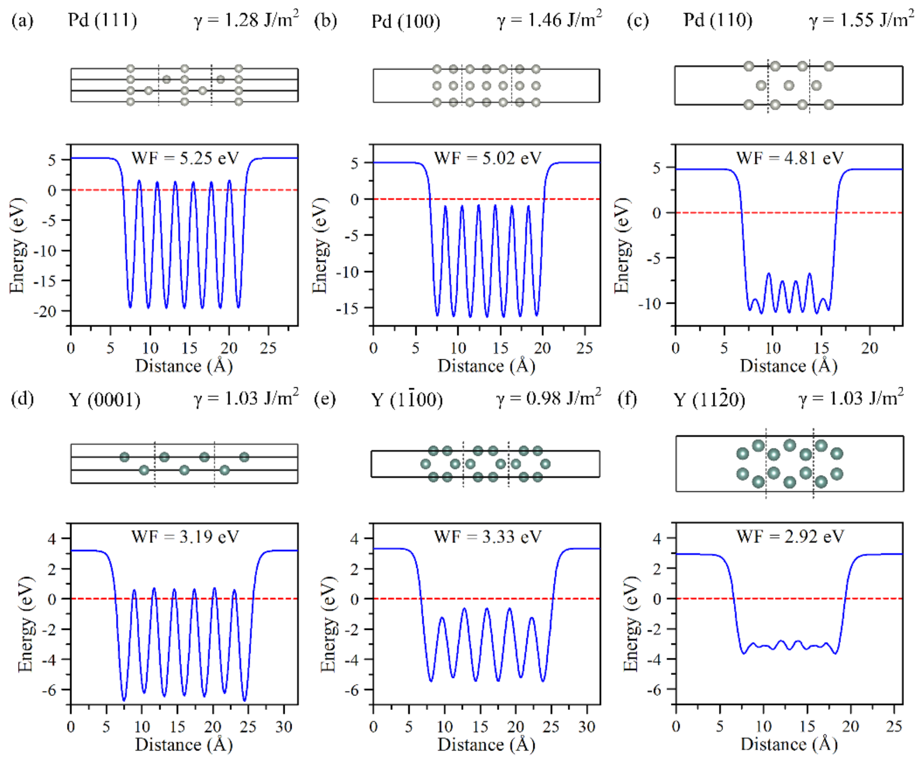

Supplementary Figure 6 | Calculated work functions of different surfaces of Y and Pd metal. The corresponding slab models and surface energies are also given. The red dashed lines indicate the Fermi levels.

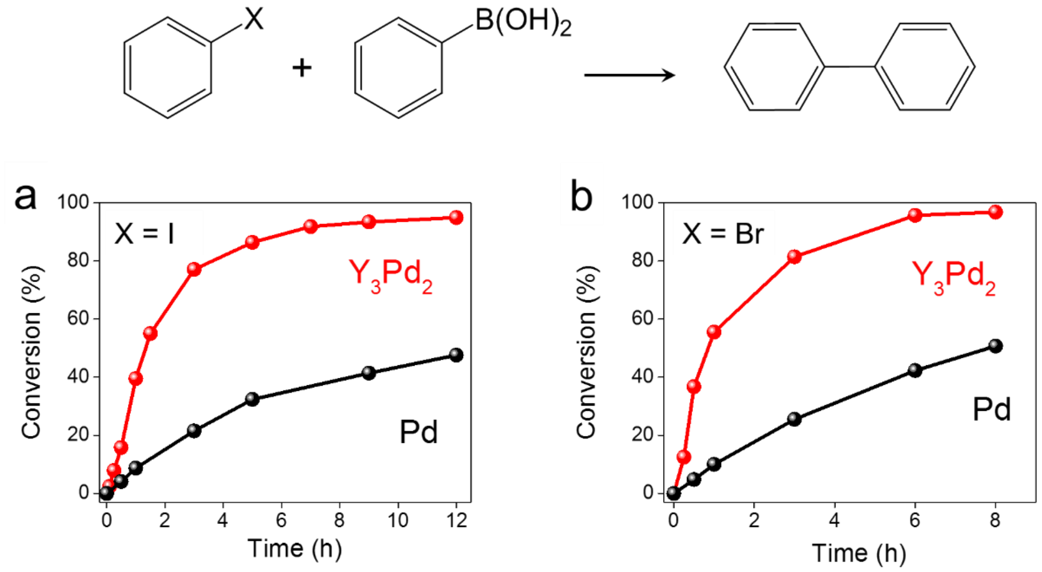

Supplementary Figure 7 Time-dependent catalysis over Y_3Pd_2 and pure Pd metal catalysts for Suzuki coupling reaction of iodobenzene and bromobenzene with phenylboronic acid. Reaction conditions: Pd (40 mol% relative to organohalide); 0.5 mmol organohalide, 0.8 mmol phenylboronic acid, 1.5 mmol K_2CO_3, 5ml solvent, iodides 30˚C, bromides 60˚C.

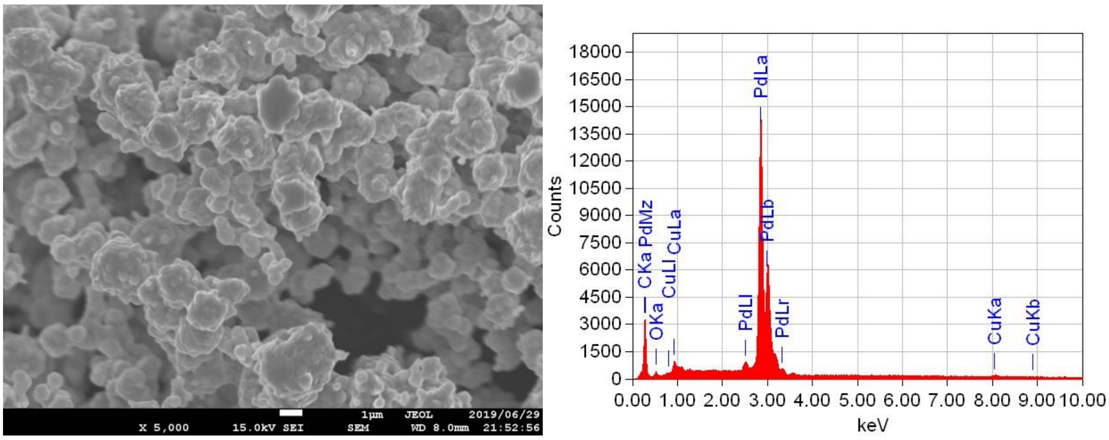

Supplementary Figure 8 | The SEM images and EDS spectra of the reference pure Pd. The signals of C and Cu are derived from carbon paste and sample holder (brass), respectively. And the tiny O species should be the adsorbed oxygen molecule due to the exposure to the air of Pd sample.

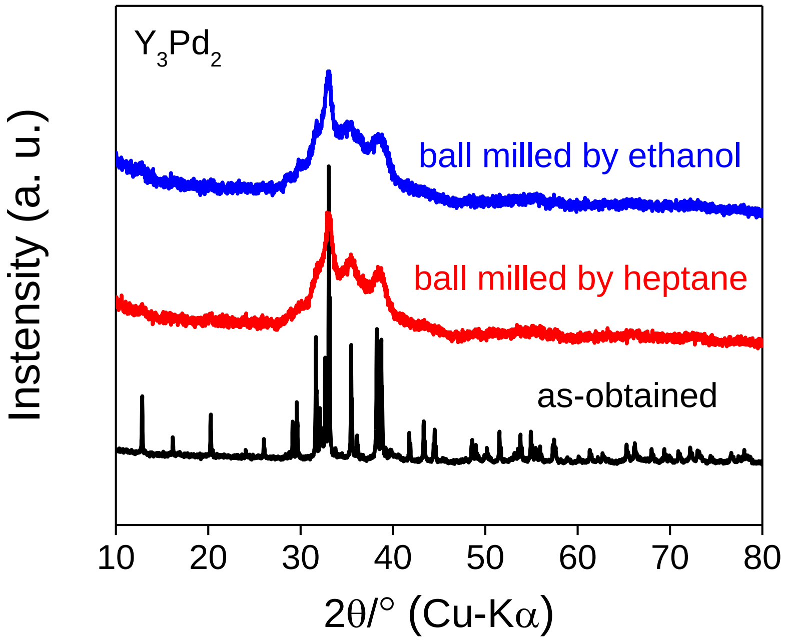

Supplementary Figure 9 | Powder XRD pattern of ball-milled Y_3Pd_2. The powder XRD pattern of the as-prepared Y_3Pd_2 and ball-milled Y_3Pd_2 in heptane and ethanol respectively. Impurity phases could not observed in the XRD patterns of both ball-milled samples compared with as-prepared one. Ball millings were conducted under Ar atmosphere with heptane or ethanol. The rotation speeds were set to 250 rpm for each and milled for 10 minutes with 10 minutes intervals for cooling. This cycle was repeated 20 times. Despite the peaks became broader after ball milling, the main peaks still can be identified in these XRD patterns.

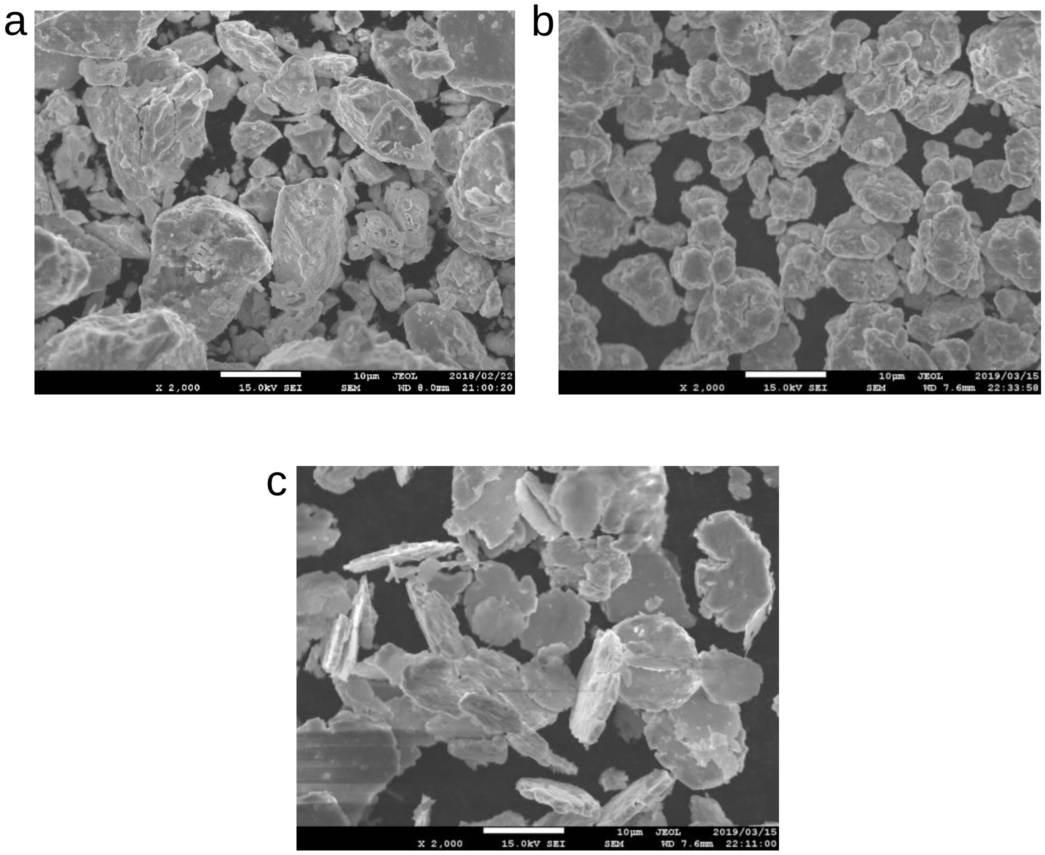

Supplementary Figure 10 The SEM images of the (a) as-prepared Y_3Pd_2 and ball-milled Y_3Pd_2 in (b) heptane and (c) ethanol respectively.

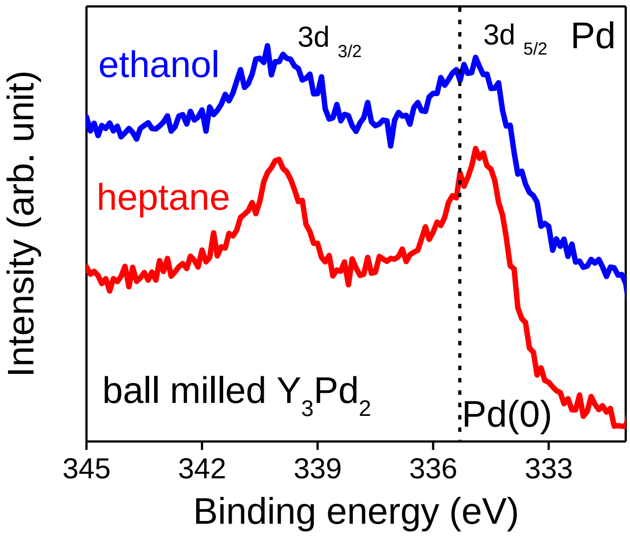

Supplementary Figure 11 XPS Pd 3*d* spectra for ball-milled Y_3Pd_2 in heptane and ethanol respectively. The XPS measurements revealed obvious red shifts of typical Pd 3*d* peaks, indicating the Pd active sites in these compounds were negative charged.

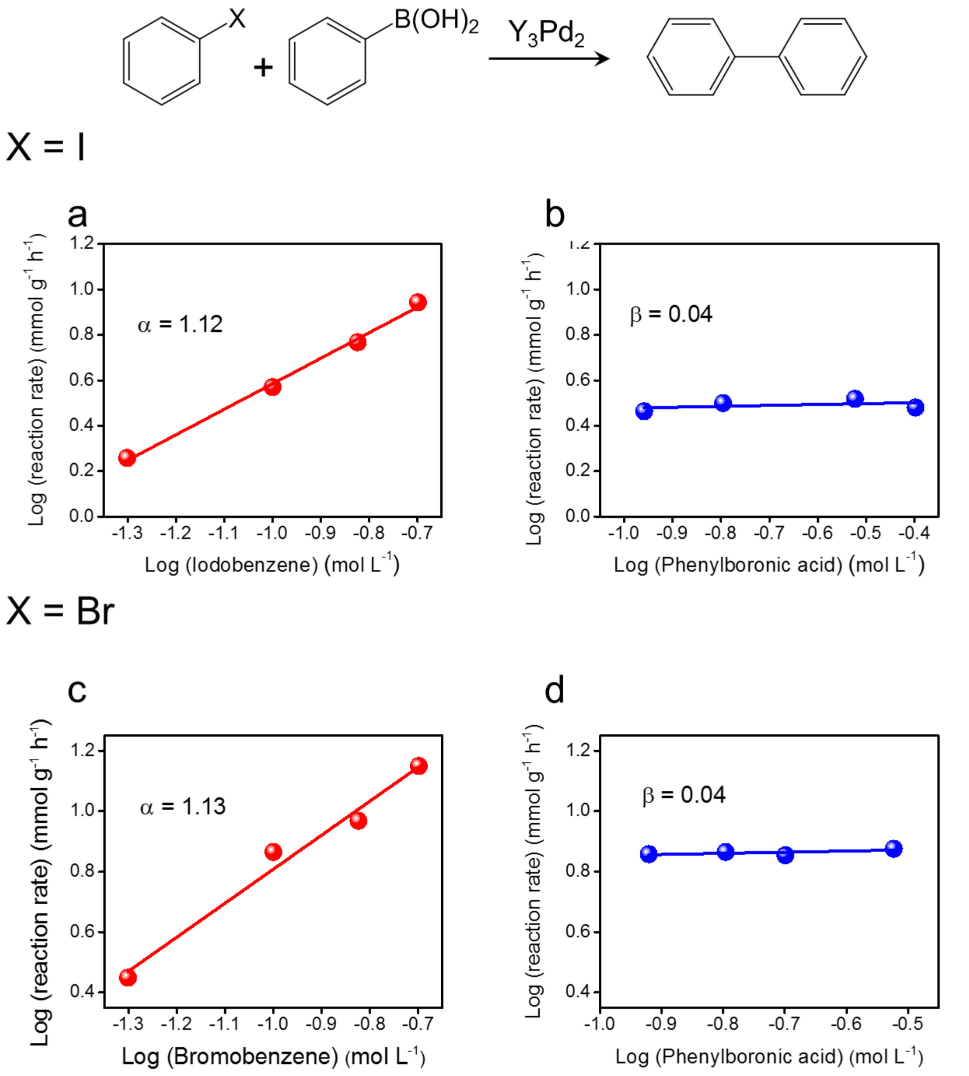

Supplementary Figure 12 a, c. Dependences of reaction rate on aryl halides (iodo and bromo) concentration over the Y_3Pd_2 catalyst. b, d. Dependences of reaction rate on phenylboronic acid concentration over the Y_3Pd_2 catalyst. Reaction conditions: Pd (40 mol% relative to organohalide), 1.5 mmol K_2CO_3, 5ml solvent, iodides 30 ˚C, bromides 60 ˚C.

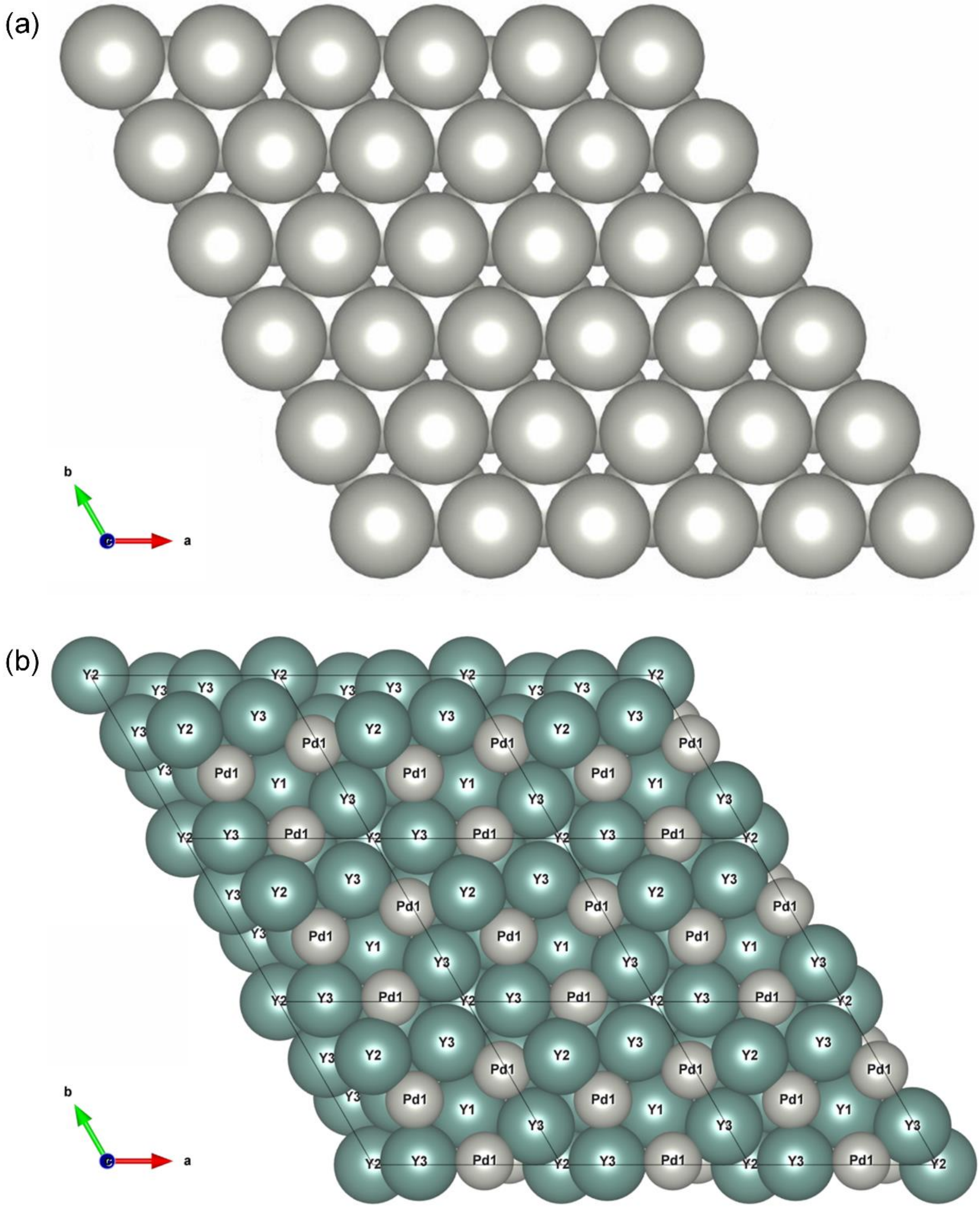

Supplementary Figure 13 Top views of (a) Pd(111) and (b) Y_3Pd_2(0001) surfaces.

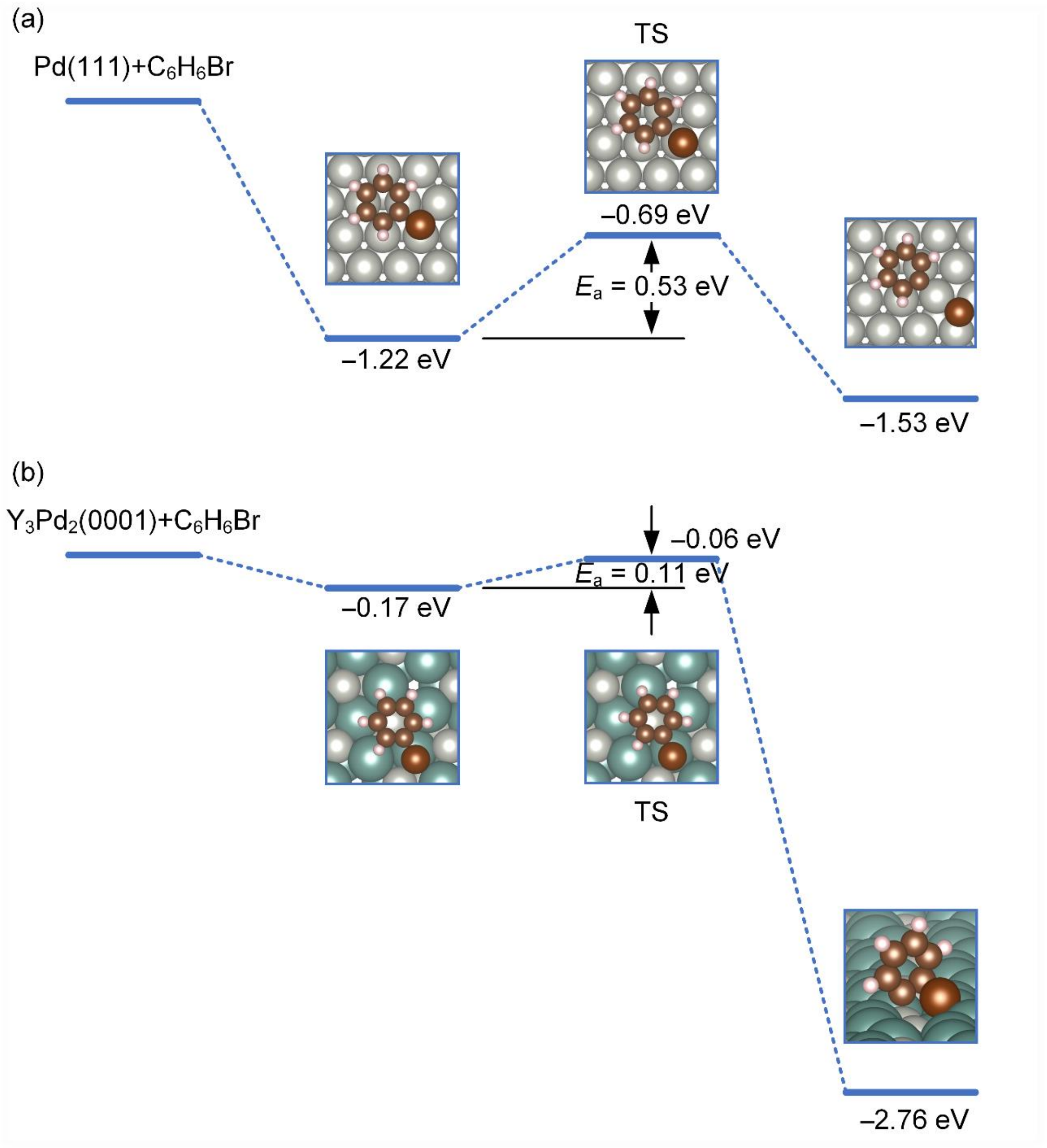

Supplementary Figure 14 Energy profiles of C_6H_5Br activation over (a) Pd(111) and (b) Y_3Pd_2(0001) surfaces.

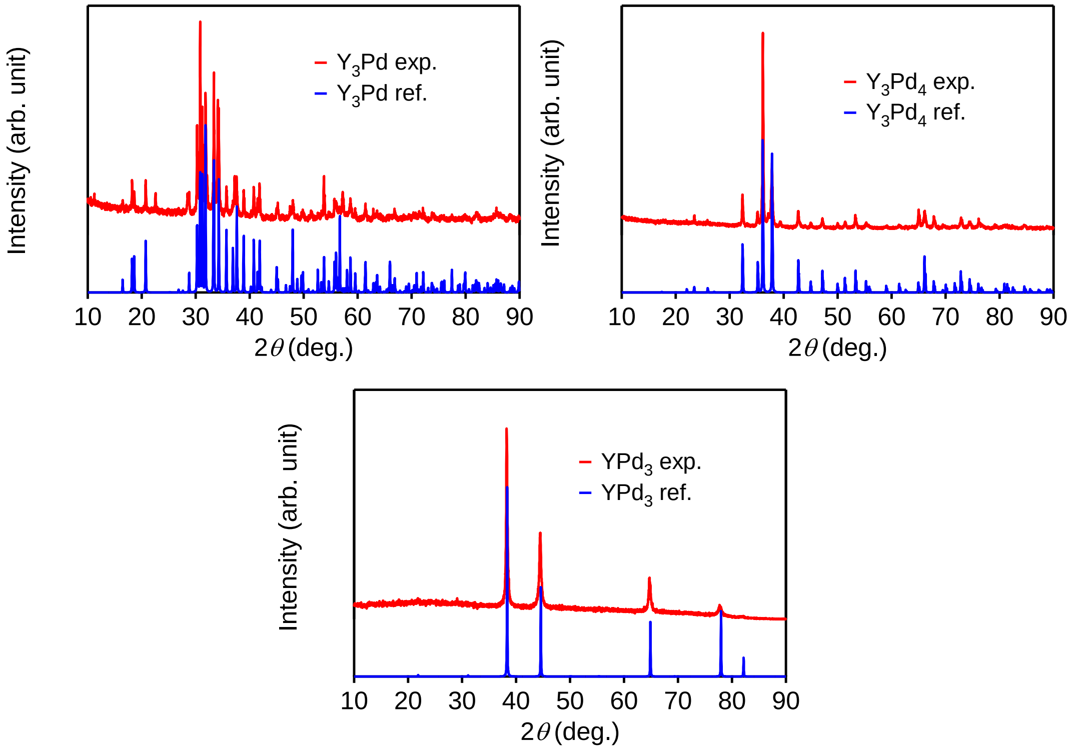

Supplementary Figure 15 | Powder XRD pattern of Y_3Pd, Y_3Pd_4 and YPd_3. The red and blue solid lines represent experimentally obtained and calculated pattern.

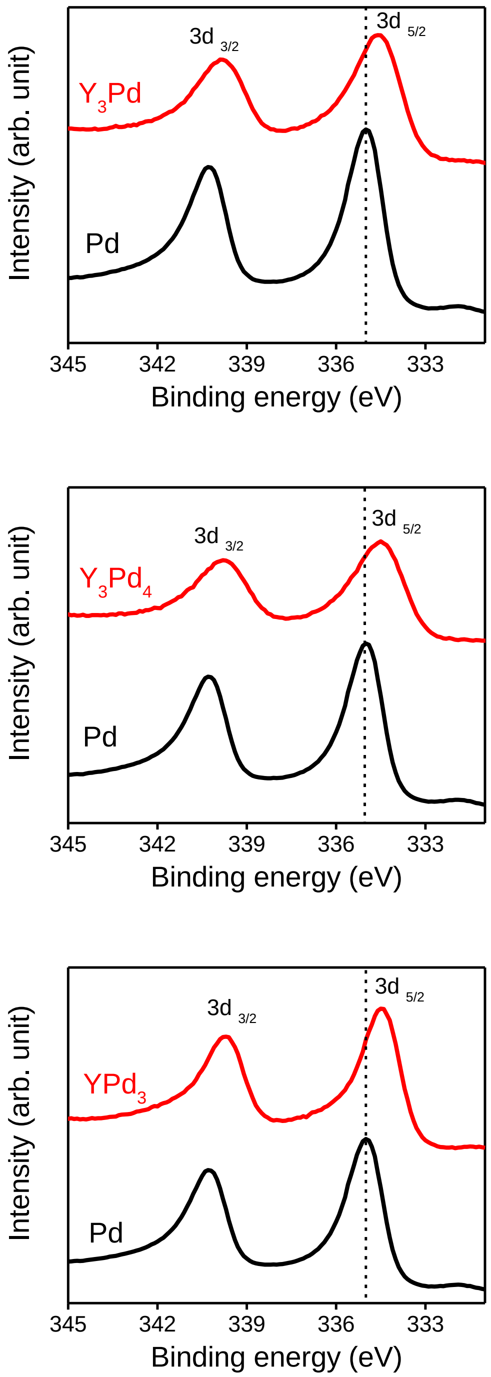

Supplementary Figure 16 | XPS Pd 3*d* spectra for the Pd, Y_3Pd, Y_3Pd_4 and YPd_3 catalyst. The XPS measurements revealed obvious red shifts of typical Pd 3d peaks, indicating the Pd active sites in these compounds were also negative charged.

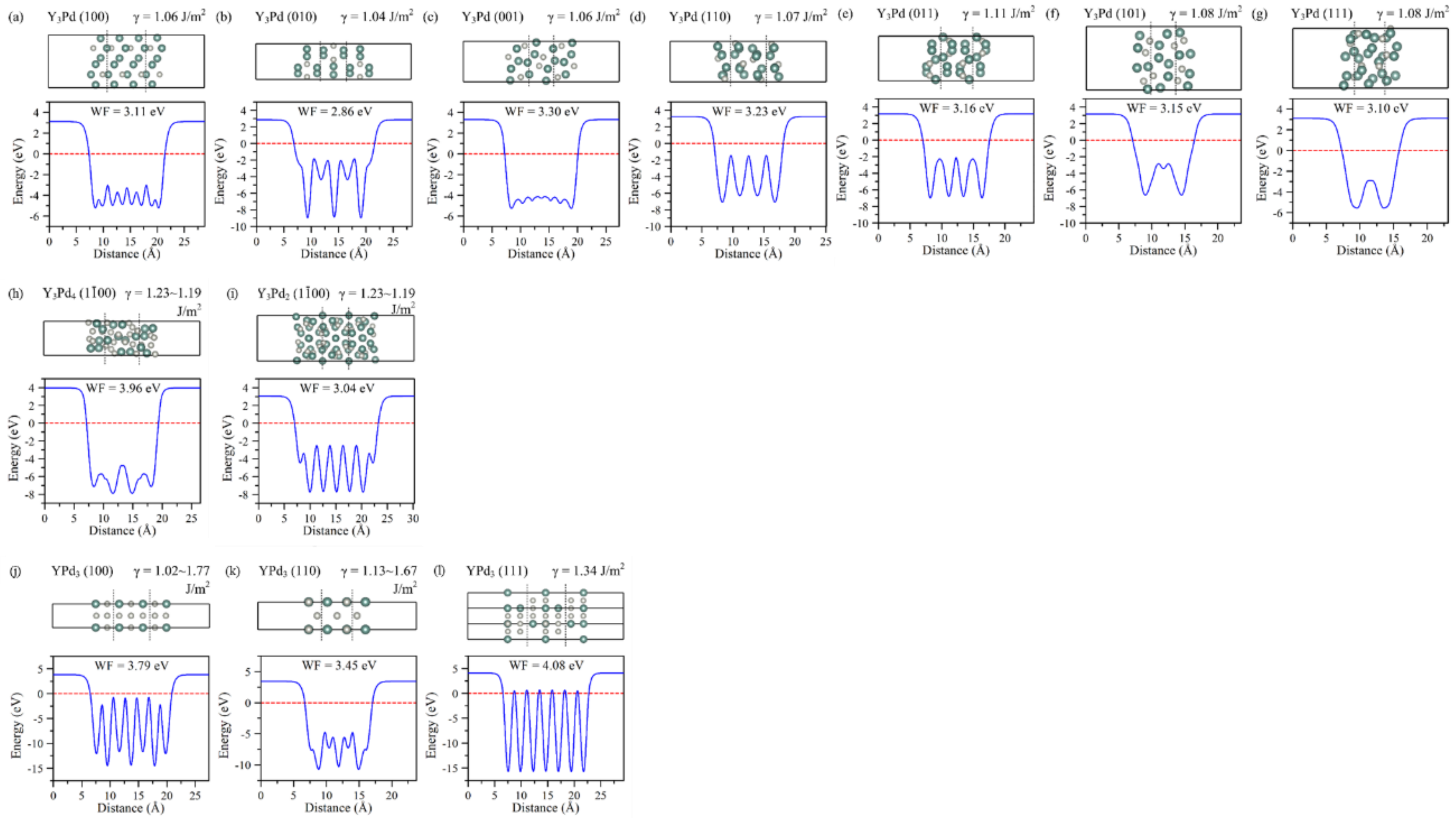

Supplementary Figure 17 | Calculated work functions of Y_3Pd, Y_3Pd_4 and YPd_3. The corresponding slab models and surface energies are also given. The red dashed lines indicate the Fermi levels.

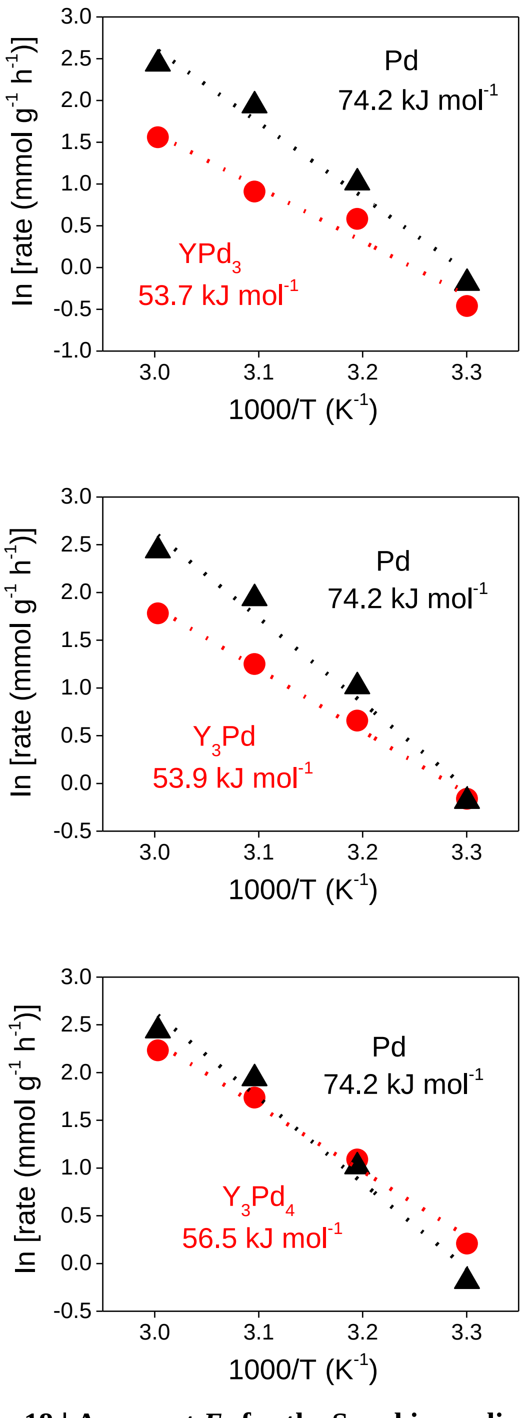

Supplementary Figure 18 | Apparent E_a for the Suzuki coupling reaction over Y_3Pd, Y_3Pd_4 and YPd_3 compounds and pure Pd metal catalysts. Reaction conditions: Pd (40 mol% relative to organohalide); 0.5 mmol iodobenzene, 0.8 mmol phenylboronic acid, 1.5 mmol K_2CO_3, 5 mL solvent. An error analysis was performed for these data and the error range was determined to be ±5%.

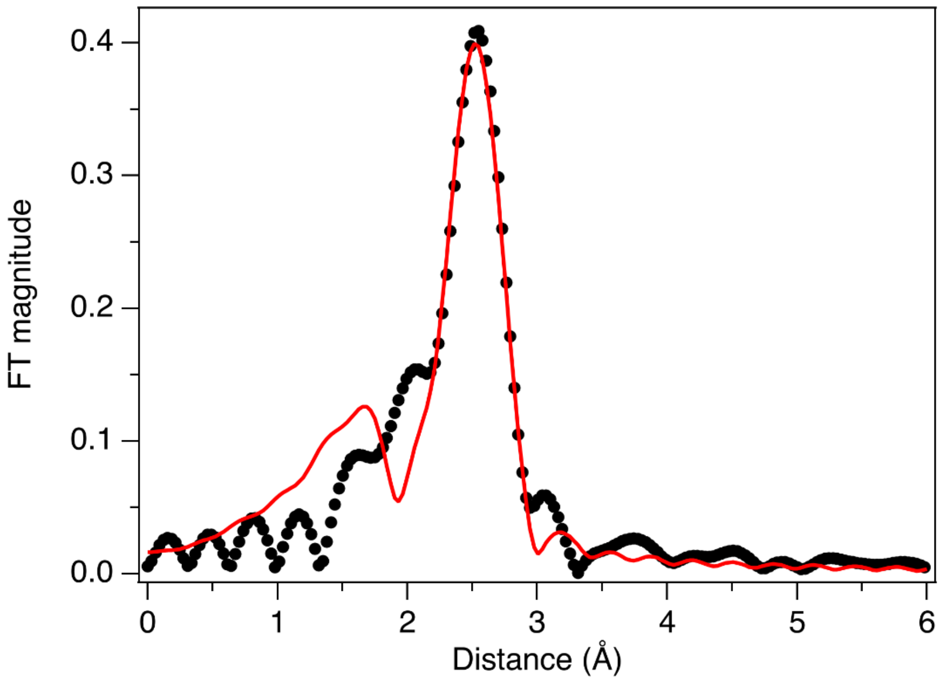

Supplementary Figure 19 | EXAFS fitting result by Y_3Pd_2 structure. The R-factor is 0.082. Black dots denote the experimental data, and red line the fitting curve.

Another additional EXAFS fitting in order to check the stoichiometry was also performed. With keeping Y_3Pd_2 geometry, the amplitudes of Pd-Pd and Pd-Y paths in the EXAFS fitting procedure were independently fitted each other. The as-fitted numbers gave $Y_{3.57}Pd_{2.09}$. If the numbers are normalized by Y number to 3, it goes to $Y_3Pd_{1.75}$. Considering fitting errors, this estimated composition is in a good agreement with the bulk 3:2 stoichiometry.

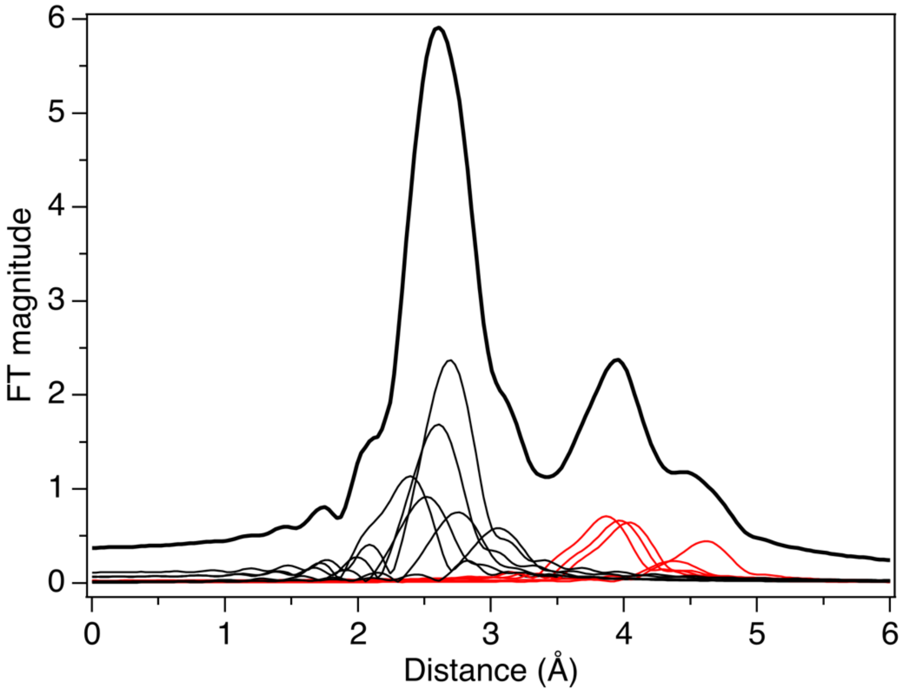

Supplementary Figure 20 | FEFF simulated amplitudes of paths for the ideal Y_3Pd_2 structure. Thin lines denote the amplitudes of the paths, and the red ones represent long range paths. All the amplitudes of the paths summed up to black bold line. Note that this black bold line does not correspond to the real EXAFS FT, because the phases of the paths should be considered. See text and Supplementary Figure 21.

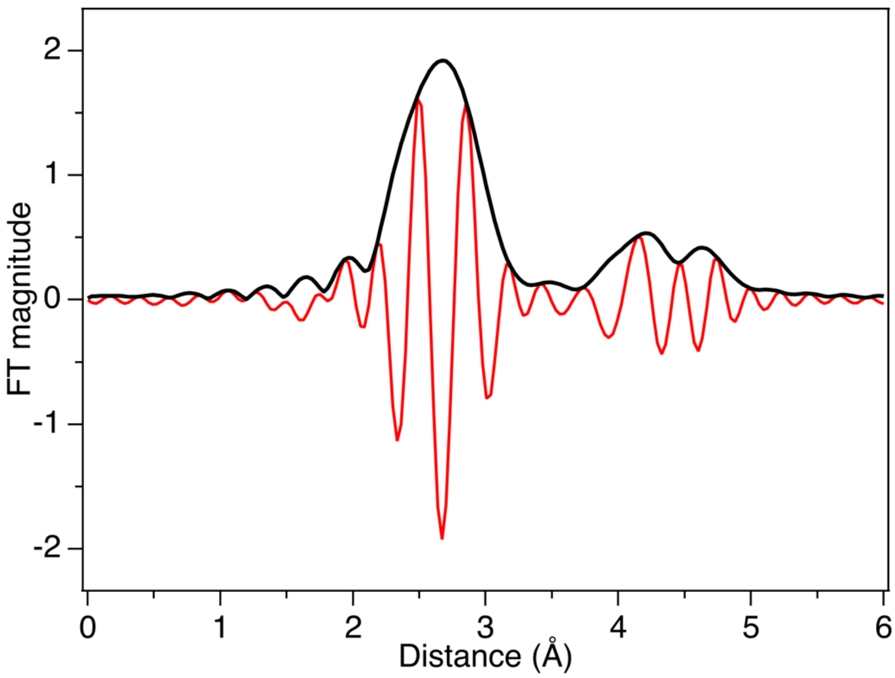

Supplementary Figure 21 The sum of the imaginary parts of the paths simulated for the ideal Y_3Pd_2 structure (red line) and its envelop function (black bold line). A few peaks are still found around 4 Å, but the intensities are not strong compared to Supplementary Figure 20. Note that this is the simulation based on the ideal Y_3Pd_2 structure and that the real structure is more or less deviated. The deviations in addition to thermal vibrations and some other effects like multiple scattering weaken long range peaks. Consequently, strong peaks in the long range were not observed in EXAFS FT (Supplementary Figure 19).

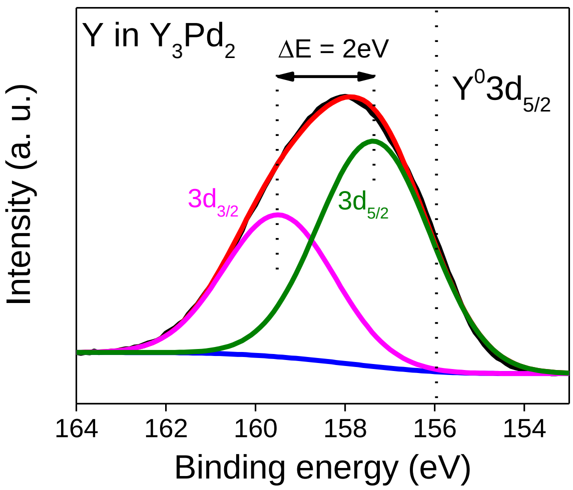

Supplementary Figure 22 | XPS analysis of Y 3*d* spectra in Y_3Pd_2. Here, Y 3*d* peaks gave a significantly shift to higher energy compared to that of Y^0 3*d*, indicating that Y is positively charged in Y_3Pd_2.

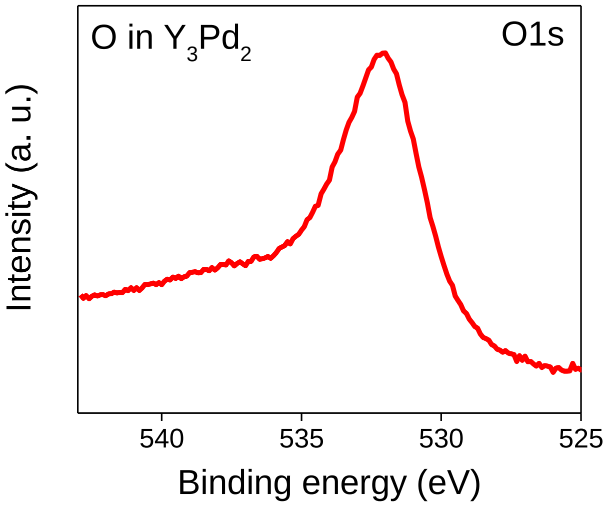

Supplementary Figure 23 | XPS analysis of O 1*s* spectra in Y_3Pd_2. It is reported that the binding energy at 529.1 eV was originated from the Y-O bond in stoichiometric Y_2O_3 film.[1] Here, O 1*s* spectra in Y_3Pd_2 gave no obvious peak at 529.1 eV compared to that of Y_2O_3, indicating that the generation of surface Y-oxide can also be ruled out.

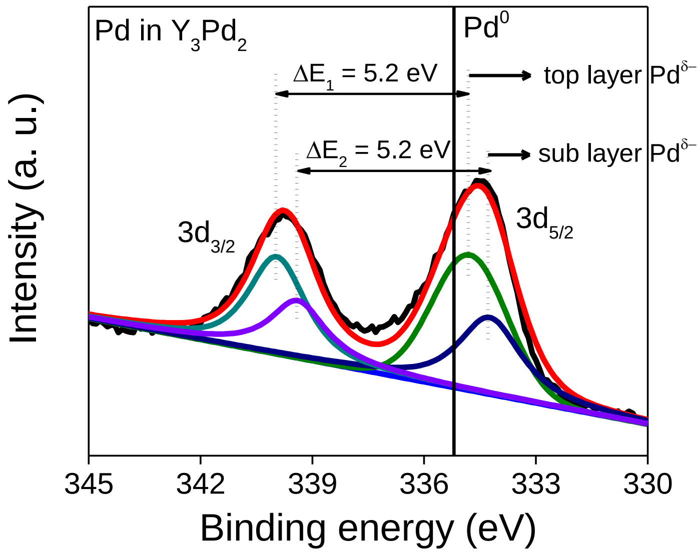

Supplementary Figure 24 | XPS analysis of Pd 3*d* spectra in Y_3Pd_2. Here, Pd 3*d* peaks gave a significantly shift to lower energy compared to that of Pd^0 3*d*, indicating that Pd is negative charged in Y_3Pd_2.

Normally, the binding energy peak position of a specific element depends on the oxidation state and local chemical environment. In the case of modifying a sample by adding other elements, if the electronegativity of the doped element (like Y in our case) is lower than the base element (like Pd in our case), the electron density around the base element (like Pd in our case) increases and the binding energy decreases. Therefore, the binding energy peak of Pd shifts negatively compared with the zero valence state Pd metal.

In Y_3Pd_2, for the top layer, Pd coordinated by lower coordination dominates the surface, which gave a negative charged Pd state with a negatively shifted binding energy. For the bulk, fully coordinated Pd species constituted the sub layer and deeper layers, resulting in a much negatively charged state with a more negatively shifted binding energy compared to the top layer Pd. This is because of the existence of more coordinated Y electron donors that can increase the electron density of those bulk Pd species. Both kinds of electronic structure of Pd states appeared in the XPS spectra with the detection limit of several atomic layer thickness of Y_3Pd_2 surface, which is in good agreement with our Bader charge analysis.

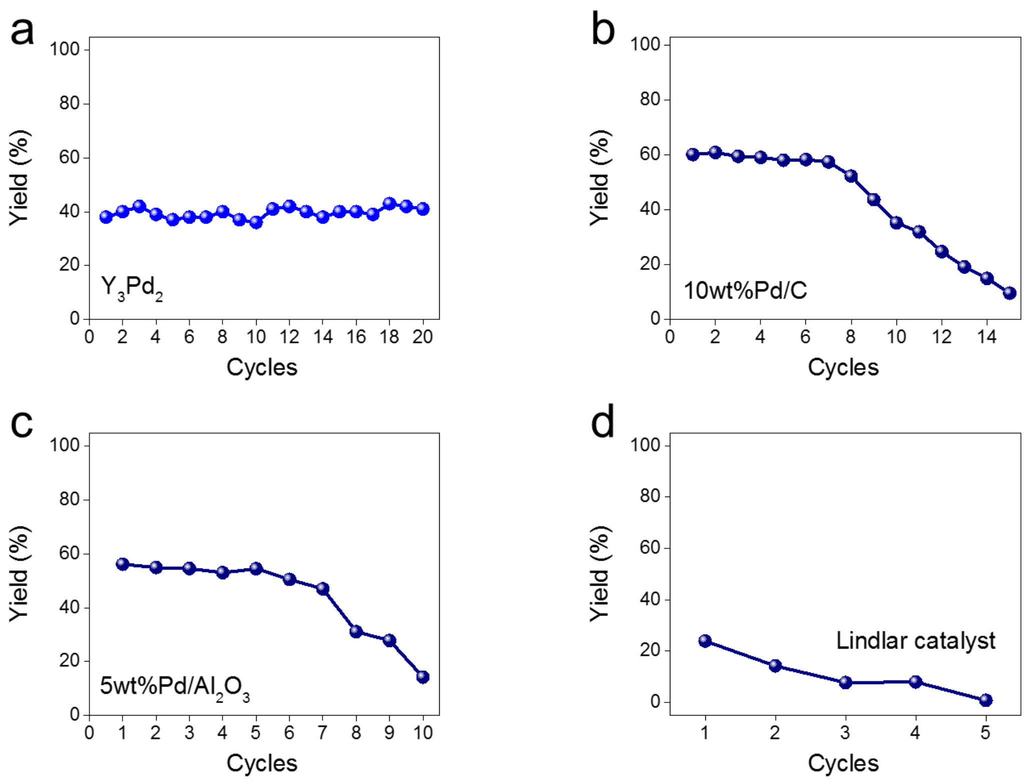

Supplementary Figure 25 Recycling experiment for the Suzuki cross-coupling reaction over a. Y_3Pd_2; b. 10wt%Pd/C; c. 5wt%Pd/Al_2O_3; d. 5wt%Pd Lindlar catalyst. Reaction conditions: Y_3Pd_2, Pd (40 mol% relative to organohalide); 10wt%Pd/C (20 mg); 5wt%Pd/Al_2O_3 (20 mg); 5wt%Pd Lindlar catalyst (20 mg); 0.5 mmol iodobenzene, 0.8 mmol phenylboronic acid, 1.5 mmol K_2CO_3, 5mL solvent, 30 °C, 1 h. An error analysis was performed for these data and the error range was determined to be ±5%.

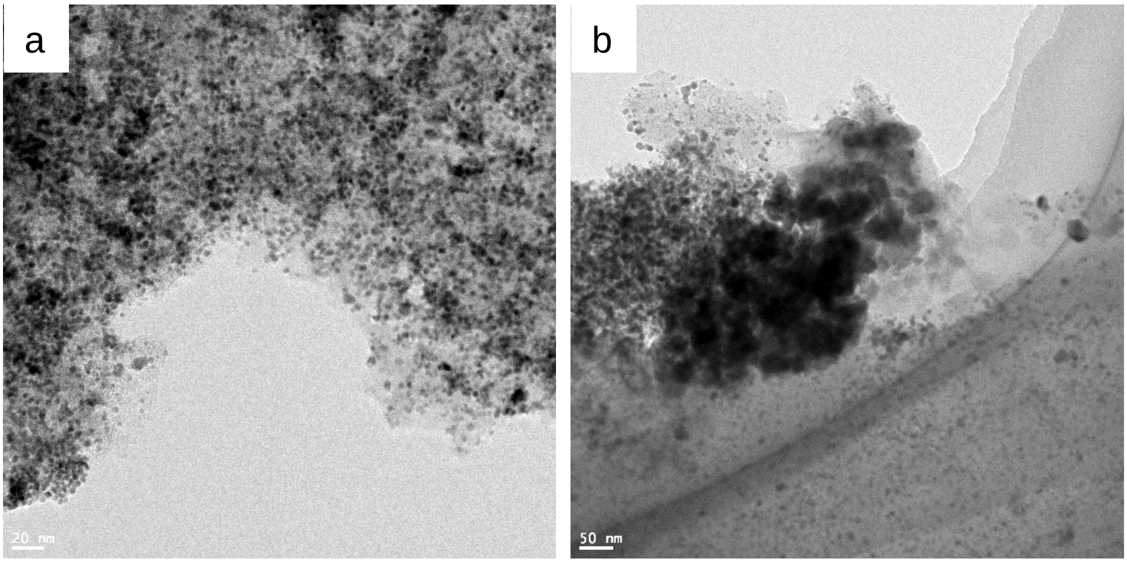

Supplementary Figure 26 TEM images of the commercial 10wt%Pd/C catalyst used as a reference **a.** before and **b.** after 15 cyclic reactions. Reaction conditions: 10wt%Pd/C (20 mg), 0.5 mmol iodobenzene, 0.8 mmol phenylboronic acid, 1.5 mmol K_2CO_3, 5mL solvent, 30 °C, 1h. Clear aggregation of Pd can be confirmed for the used catalyst.

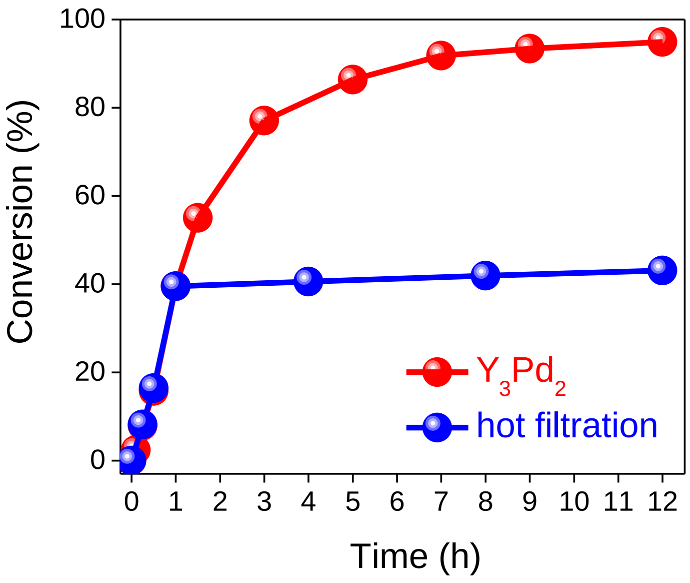

Supplementary Figure 27 | Hot filtration test of Y_3Pd_2 for Suzuki coupling reaction. The reaction no longer proceeds after removal of the Y_3Pd_2 catalyst. Reaction conditions: Pd (40 mol% relative to organohalide); 0.5 mmol iodides, 0.8 mmol phenylboronic acid, 1.5 mmol K_2CO_3, 5ml solvent, 30 °C.

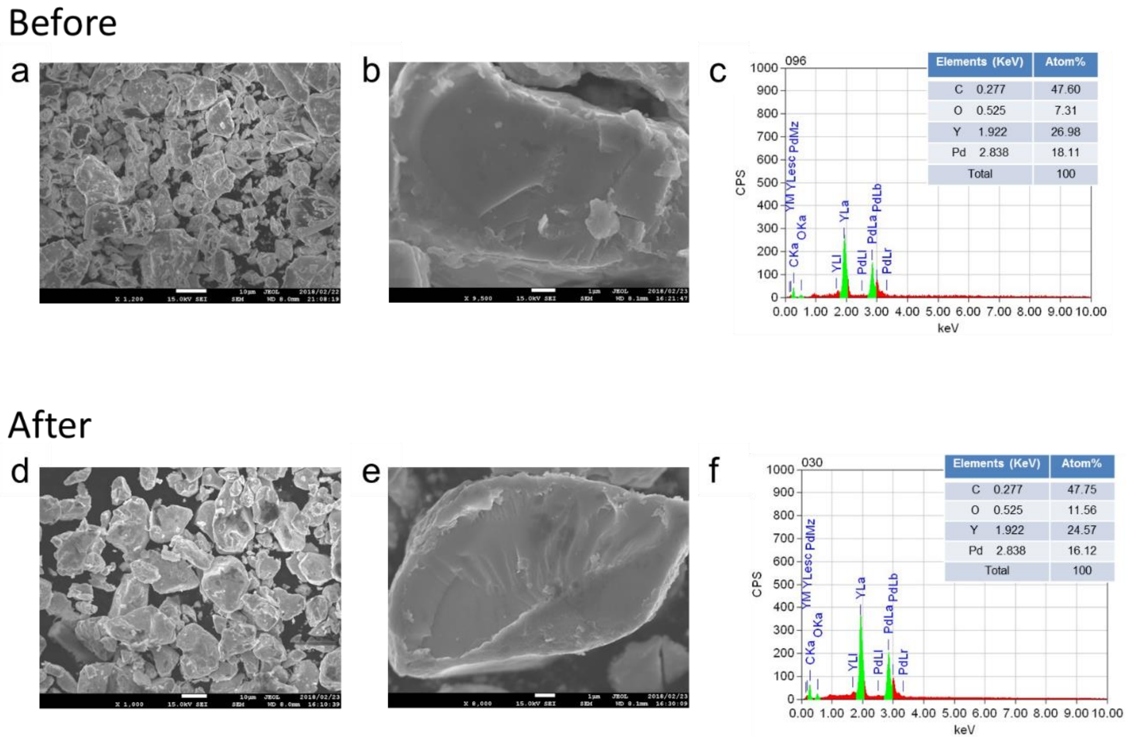

Supplementary Figure 28 SEM images and EDS spectra of the as-prepared and used Y_3Pd_2 catalyst.

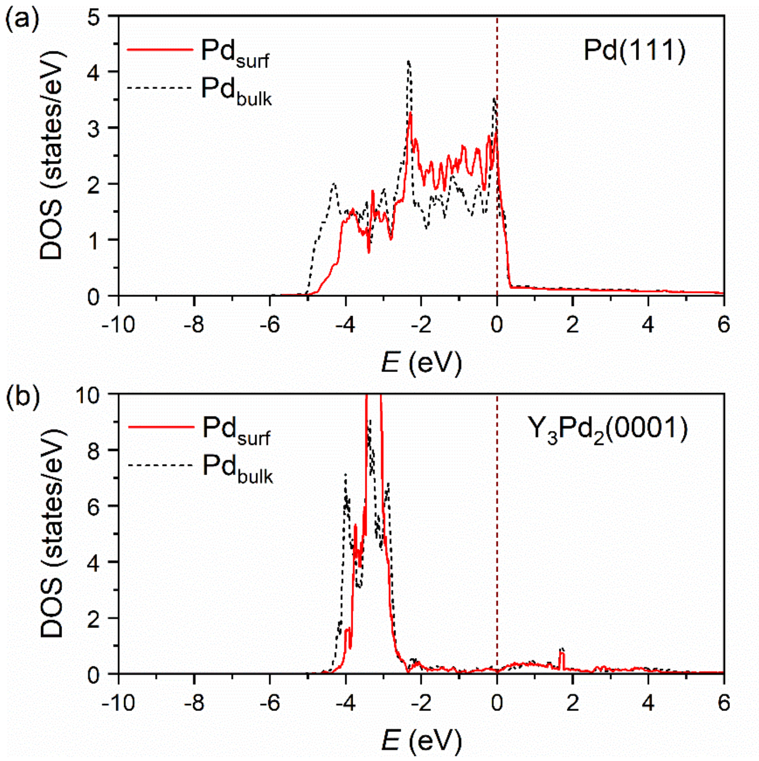

Supplementary Figure 29 Projected Pd *d*-band densities of states (DOS). The *d*-band center (ε_d) is at –0.95, –1.23, –2.31, and –2.30 eV for the Pd atoms on the surface and in the bulk of Pd(111) and Y_3Pd_2(0001), respectively.

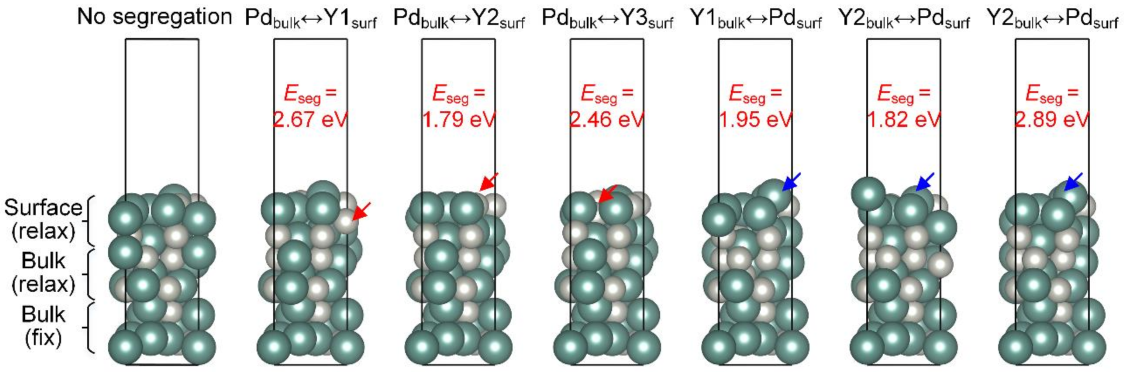

Supplementary Figure 30 Side view of the relaxed Y_3Pd_2(0001) surface structures without and with Pd and Y segregations. The Pd atoms marked with red arrow are the ones segregated from the bulk to the different Y atoms sites on the surface. The Y atoms marked with blue arrow are the ones segregated from the bulk to the Pd site on the surface. The segregation energies (E_{seg}) of Pd and Y atoms are 1.79–2.67 and 1.82–2.89 eV, respectively.

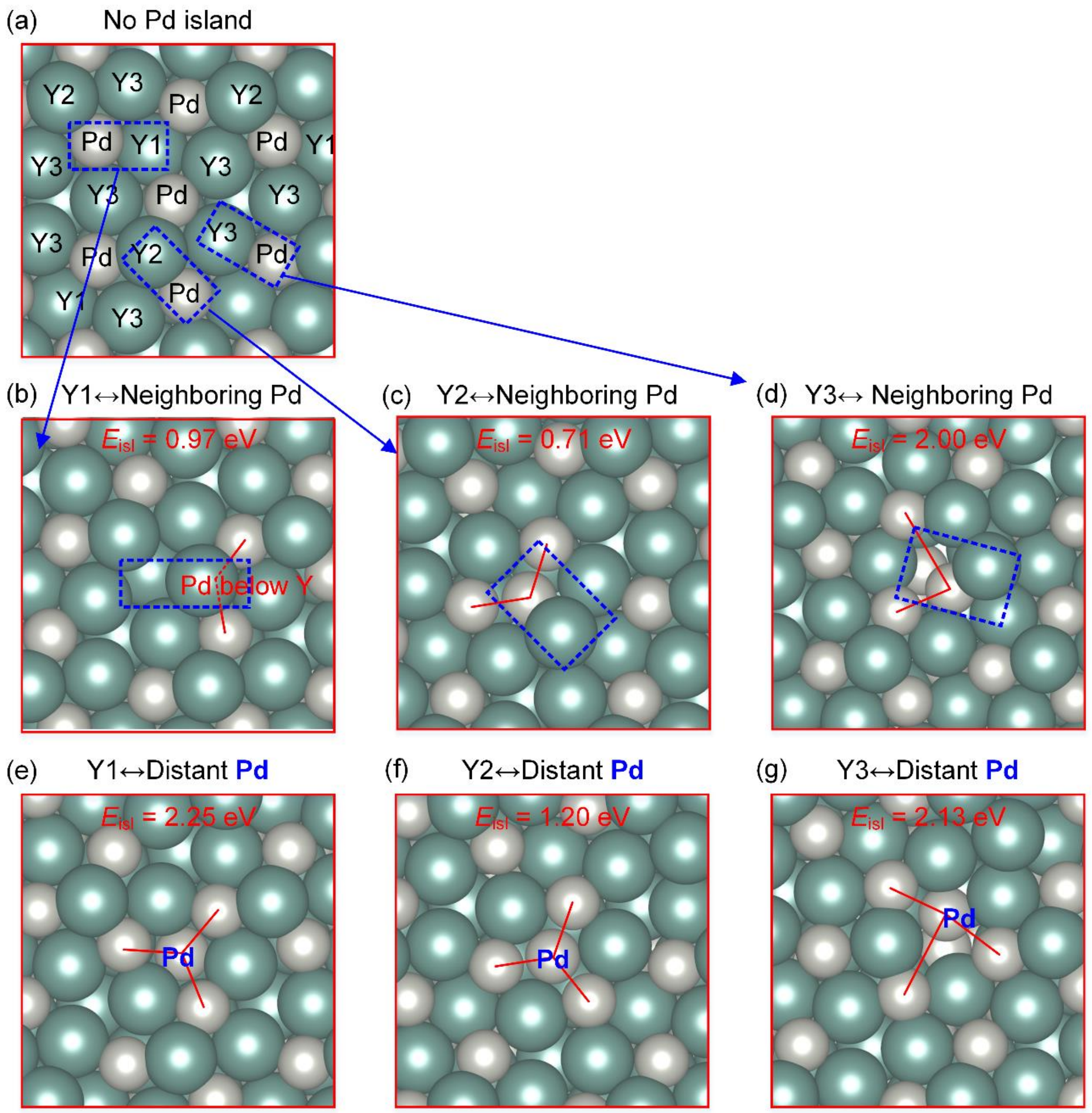

Supplementary Figure 31 Top view of the relaxed surface structures without and with Pd islands: (a) No island; (b–c) Islands formed by replacing Y1, Y2, Y3 with a neighboring Pd atom; (e–f) Islands formed by replacing Y1, Y2, Y3 atoms with a distant Pd atom. The island formation energies (E_{isl}) of Pd range from 0.71 to 2.25 eV.

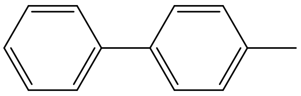

^{1}H NMR ($CDCl_3$, 400 MHz): δ = 7.56 (d, 2H), 7.48 (d, 2H), 7.40 (t, 2H), 7.30 (t, 1H), 7.23 (d, 2H), 2.38 (s, 3H); ^{13}C NMR ($CDCl_3$, 100 MHz): δ = 141.2, 138.4, 137.1, 129.5, 128.8, 127.0, 21.1.

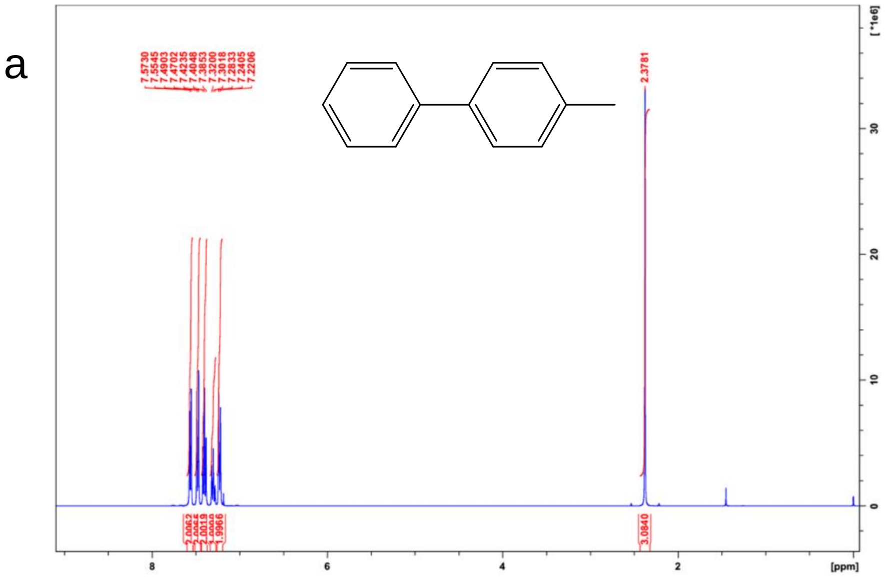

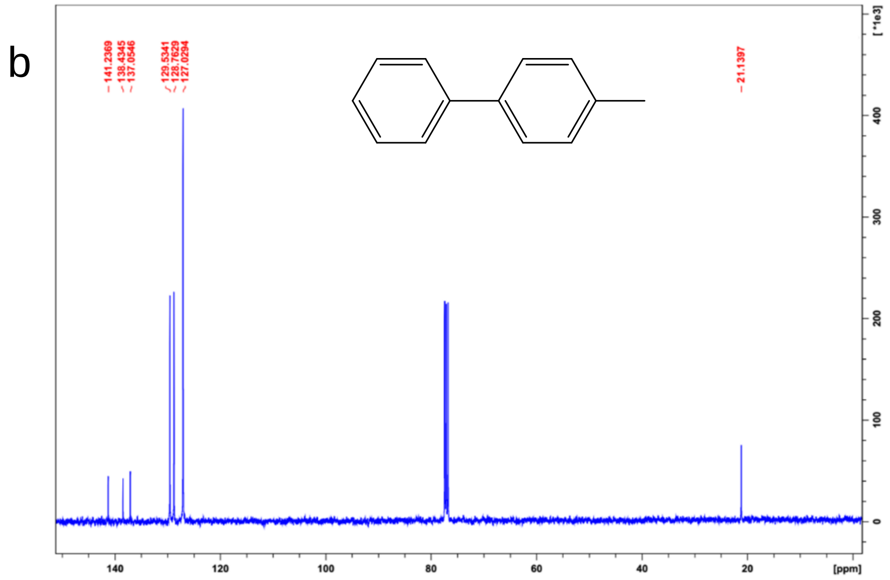

Supplementary Figure 32 a. ^{1}H NMR and b. ^{13}C NMR spectra.

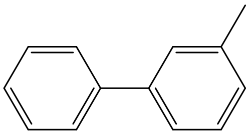

^1H NMR ($CDCl_3$, 400 MHz): δ = 7.57 (d, 2H), 7.45-7.35 (m, 4H), 7.31 (t, 2H), 7.15 (d, 1H), 2.40 (s, 3H); ^{13}C NMR ($CDCl_3$, 100 MHz): δ = 141.4, 141.3, 138.4, 128.7, 128.0, 127.2, 124.3, 21.6.

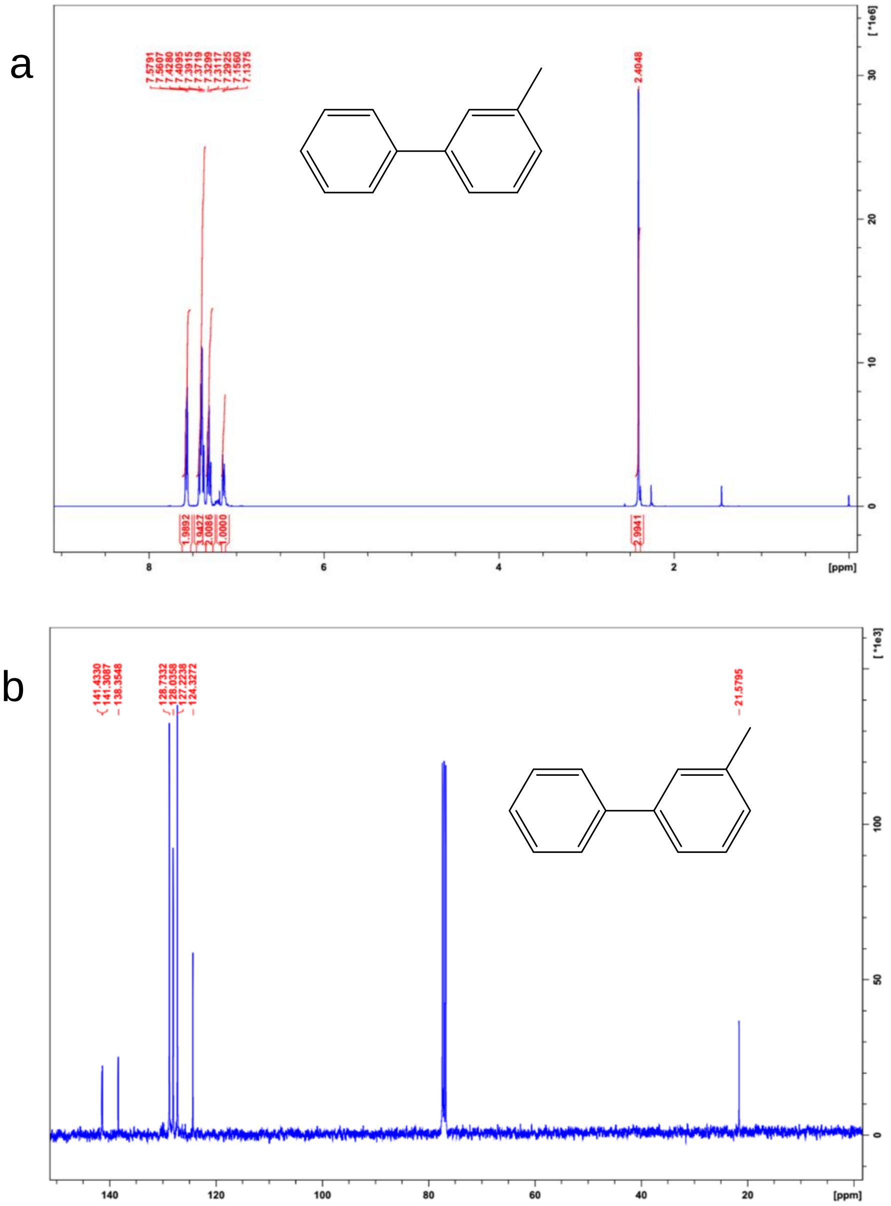

Supplementary Figure 33 a. ^1H NMR and b. ^{13}C NMR spectra.

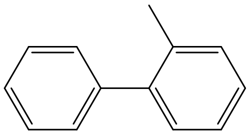

^{1}H NMR ($CDCl_3$, 400 MHz): δ = 7.40 (t, 2H), 7.38-7.29 (m, 3H), 7.27-7.21 (m, 4H), 2.26 (s, 3H); ^{13}C NMR ($CDCl_3$, 100 MHz): δ = 142.0, 135.4, 130.3, 129.8, 129.2, 128.1, 127.3, 126.8, 125.8, 20.5.

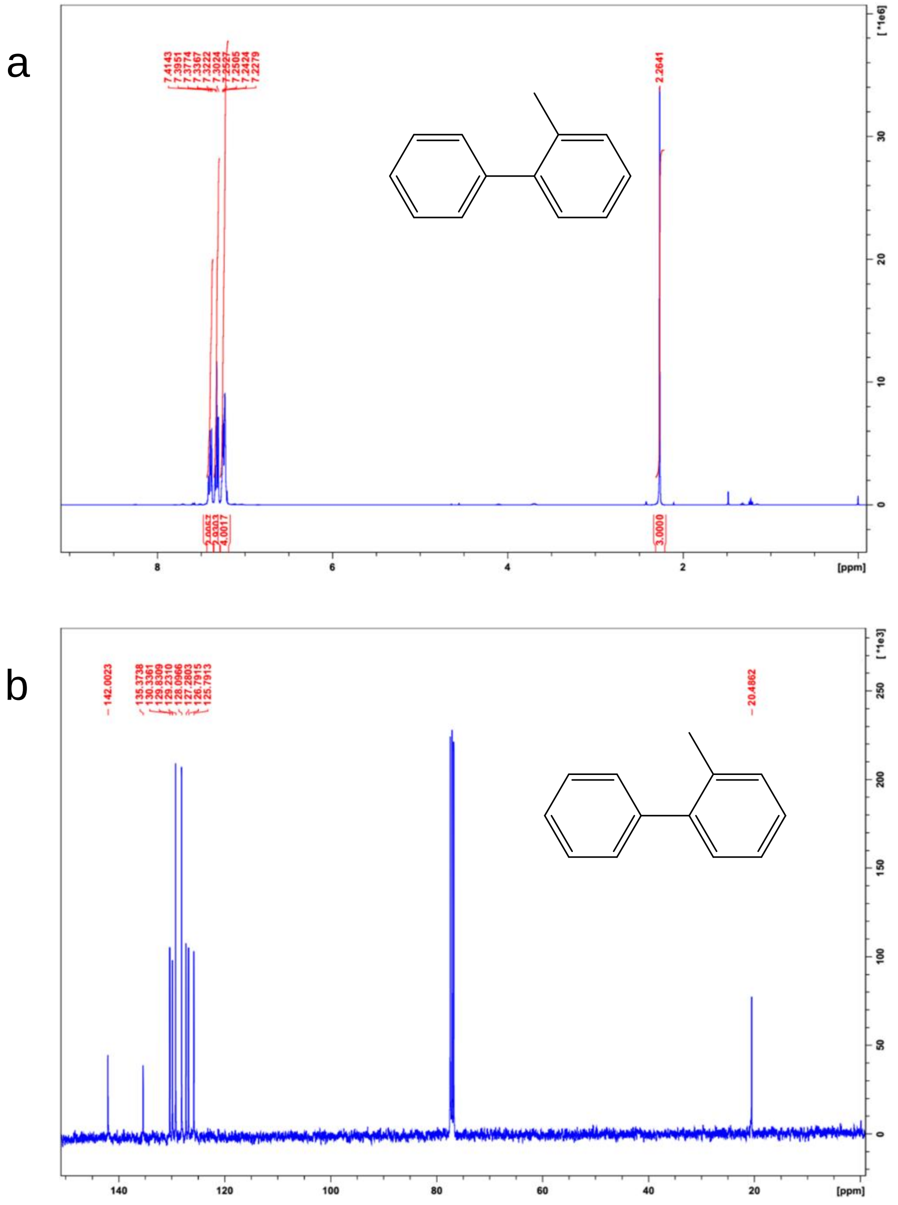

Supplementary Figure 34 a. ^{1}H NMR and b. ^{13}C NMR spectra.

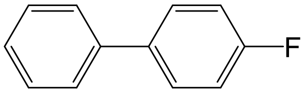

^{1}H NMR ($CDCl_3$, 400 MHz): δ = 7.56-7.48 (m, 4H), 7.42 (t, 2H), 7.32 (t, 1H), 7.11 (t, 2H); ^{13}C NMR ($CDCl_3$, 100 MHz): δ = 140.3, 137.4, 128.8, 128.7, 128.6, 127.3, 127.0, 115.7, 115.5.

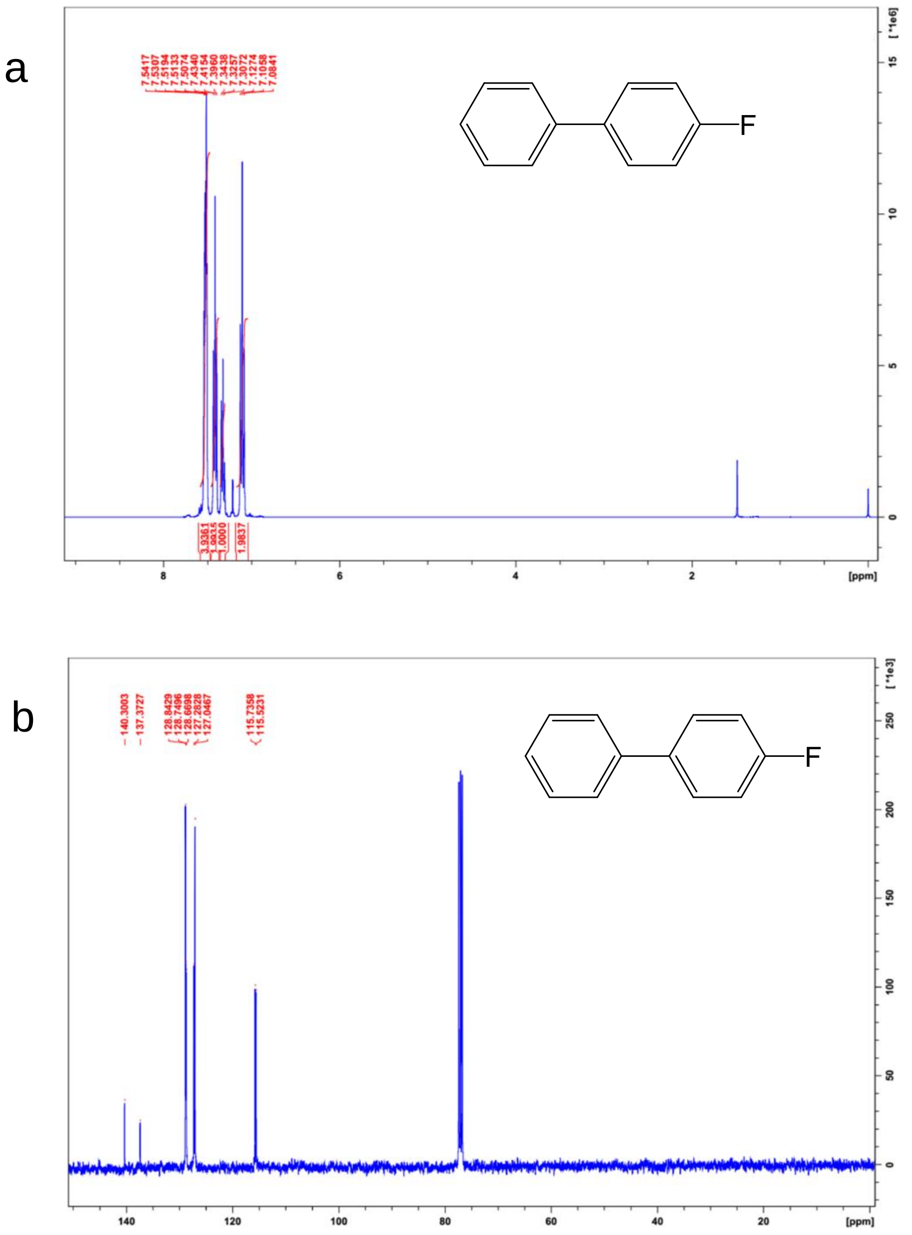

Supplementary Figure 35 a. ^{1}H NMR and b. ^{13}C NMR spectra.

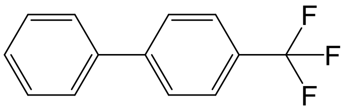

^{1}H NMR ($CDCl_3$, 400 MHz): δ = 7.68 (s, 4H), 7.58 (d, 2H), 7.49-7.35 (m, 3H); ^{13}C NMR ($CDCl_3$, 100 MHz): δ = 144.7, 139.7, 129.0, 128.2, 127.4, 127.3, 125.7, 125.6.

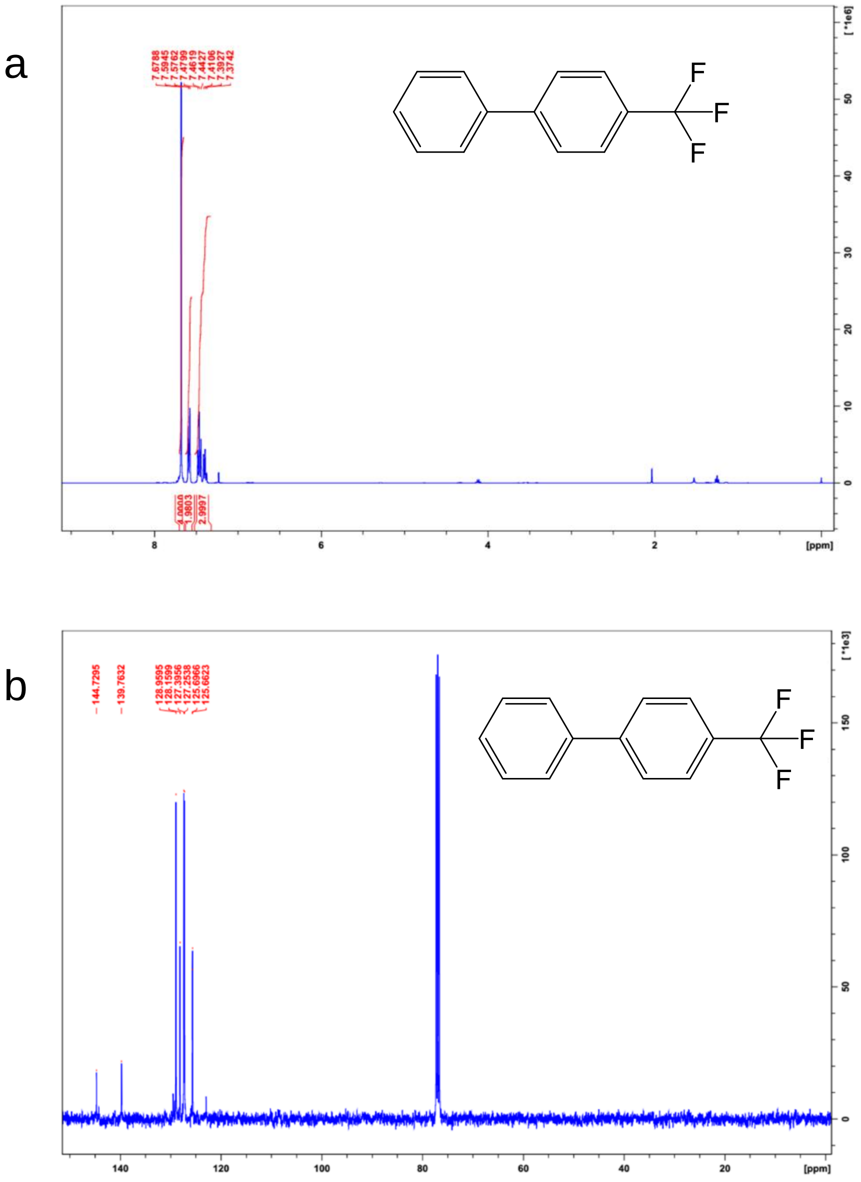

Supplementary Figure 36 a. ^{1}H NMR and b. ^{13}C NMR spectra.

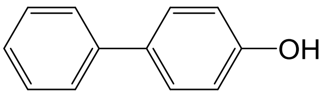

^1H NMR [$(CD_3)_2CO$, 400 MHz]: δ = 8.41 (s, 1H), 7.57 (d, 2H), 7.50 (d, 2H), 7.40 (t, 2H), 7.27 (t, 1H), 6.94 (d, 2H); ^{13}C NMR [$(CD_3)_2CO$, 100 MHz]: δ = 157.1, 141.0, 132.3, 128.7, 127.9, 126.3, 115.7.

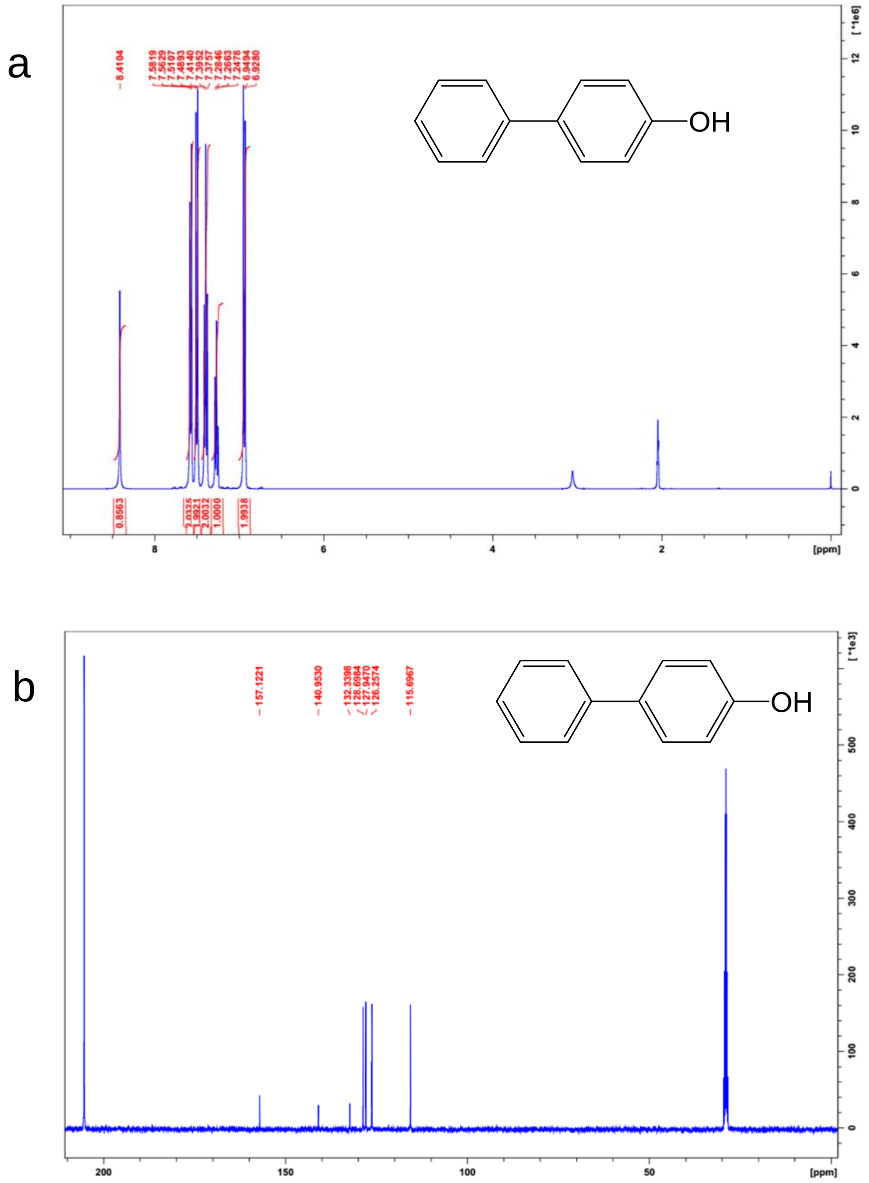

Supplementary Figure 37 a. ^1H NMR and b. ^{13}C NMR spectra.

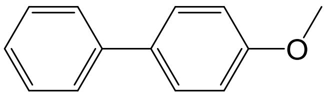

^{1}H NMR ($CDCl_3$, 400 MHz): δ = 7.53 (t, 4H), 7.40 (t, 2H), 7.28 (t, 1H), 6.96 (d, 2H), 3.82 (s, 3H); ^{13}C NMR ($CDCl_3$, 100 MHz): δ = 159.2, 140.9, 133.8, 128.8, 128.2, 126.8, 114.3, 55.4.

a

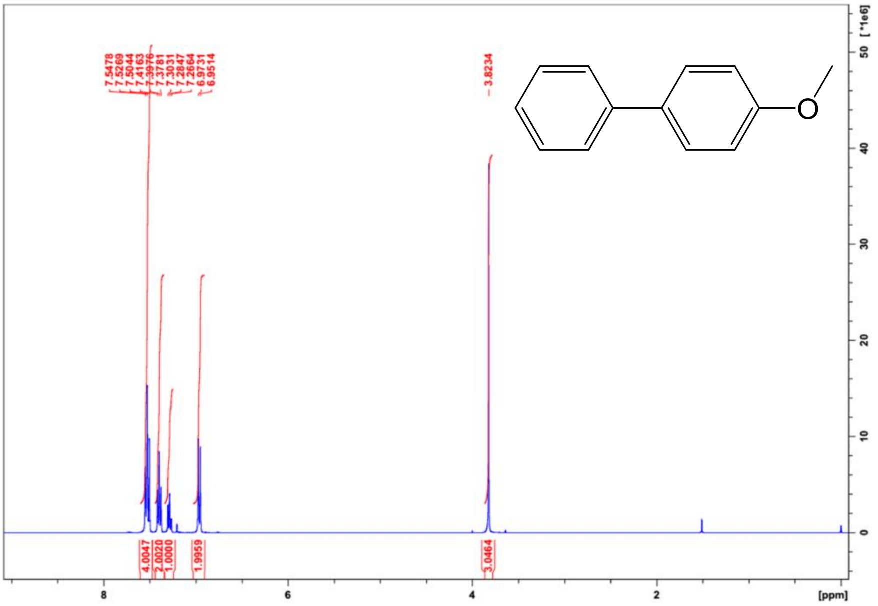

b

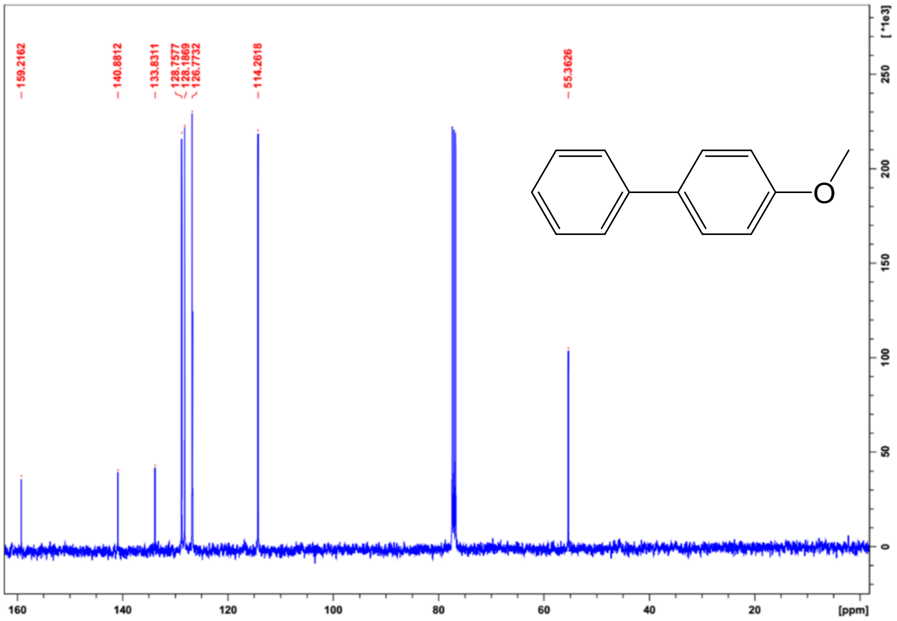

Supplementary Figure 38 a. ^{1}H NMR and b. ^{13}C NMR spectra.

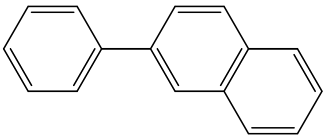

^{1}H NMR ($CDCl_3$, 400 MHz): δ = 8.02 (s, 1H), 7.91-7.83 (m, 3H), 7.83-7.69 (m, 3H), 7.50-7.37 (m, 4H), 7.38-7.33 (m, 1H); ^{13}C NMR ($CDCl_3$, 100 MHz): δ = 141.2, 138.6, 133.8, 132.7, 128.9, 128.5, 128.3, 127.7, 127.5, 127.4, 126.3, 126.0, 125.9, 125.6.

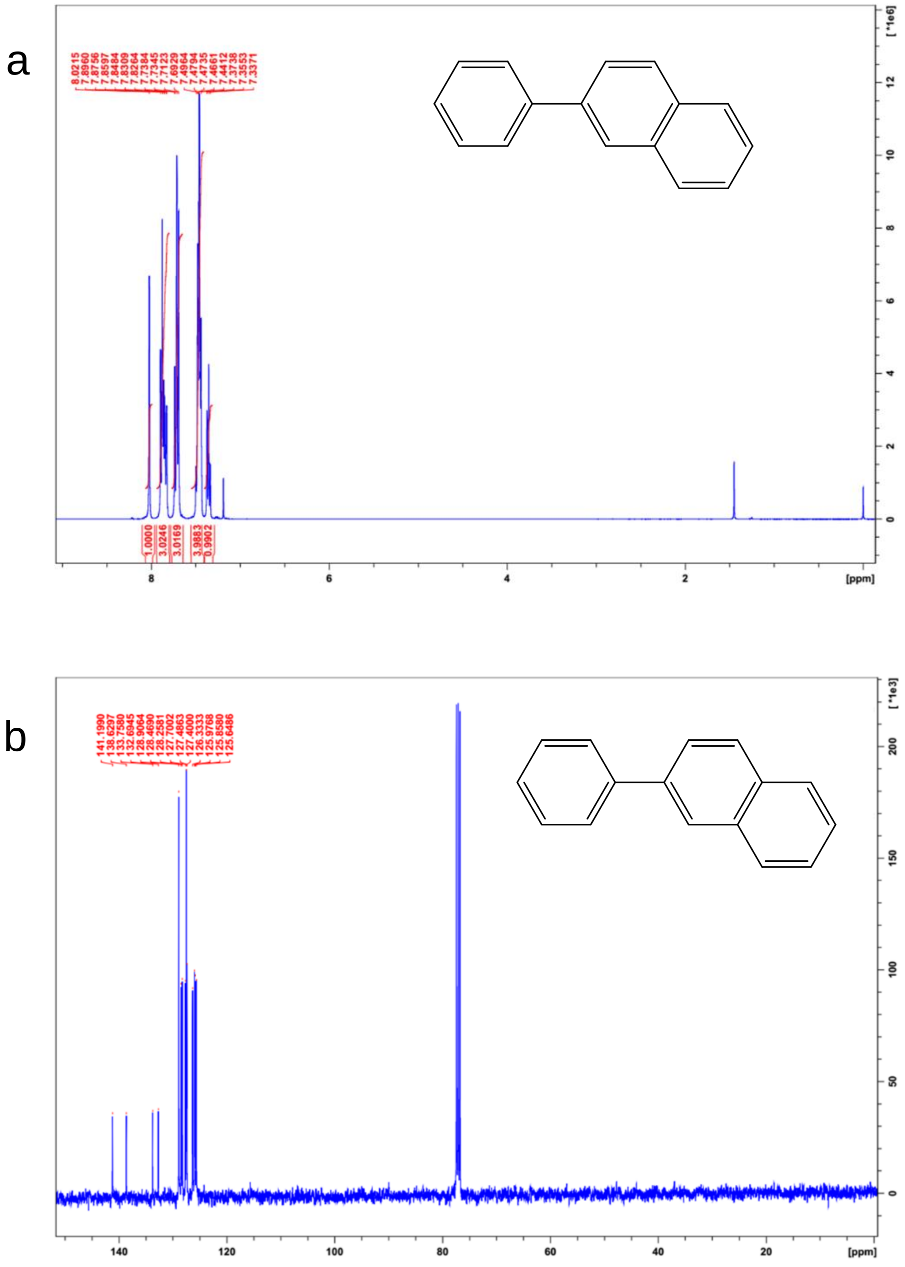

Supplementary Figure 39 a. ^{1}H NMR and b. ^{13}C NMR spectra.

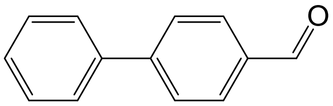

^{1}H NMR ($CDCl_3$, 400 MHz): δ = 10.0 (s, 1H), 7.94 (d, 2H), 7.74 (d, 2H), 7.62 (d, 2H), 7.49-7.38 (m, 3H); ^{13}C NMR ($CDCl_3$, 100 MHz): δ = 191.9, 147.2, 139.7, 135.3, 130.3, 129.0, 128.5, 127.7, 127.4.

a

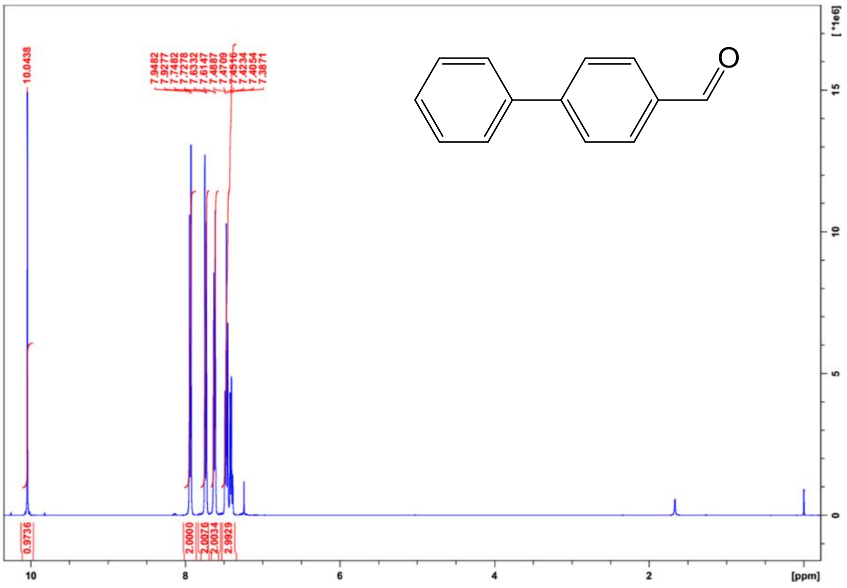

b

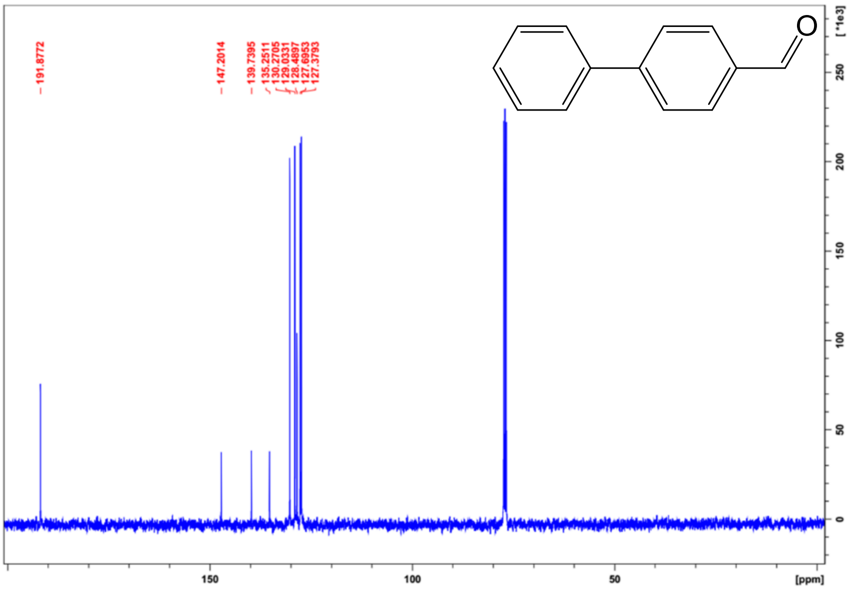

Supplementary Figure 40 a. ^{1}H NMR and b. ^{13}C NMR spectra.

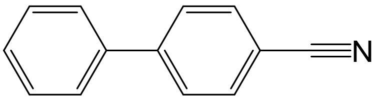

^{1}H NMR ($CDCl_3$, 400 MHz): δ = 7.72-7.65 (m, 4H), 7.59-7.56 (d, 2H), 7.50-7.39 (m, 3H); ^{13}C NMR ($CDCl_3$, 75 MHz): δ = 145.7, 139.2, 132.6, 129.1, 128.7, 127.7, 127.2, 118.9, 111.0.

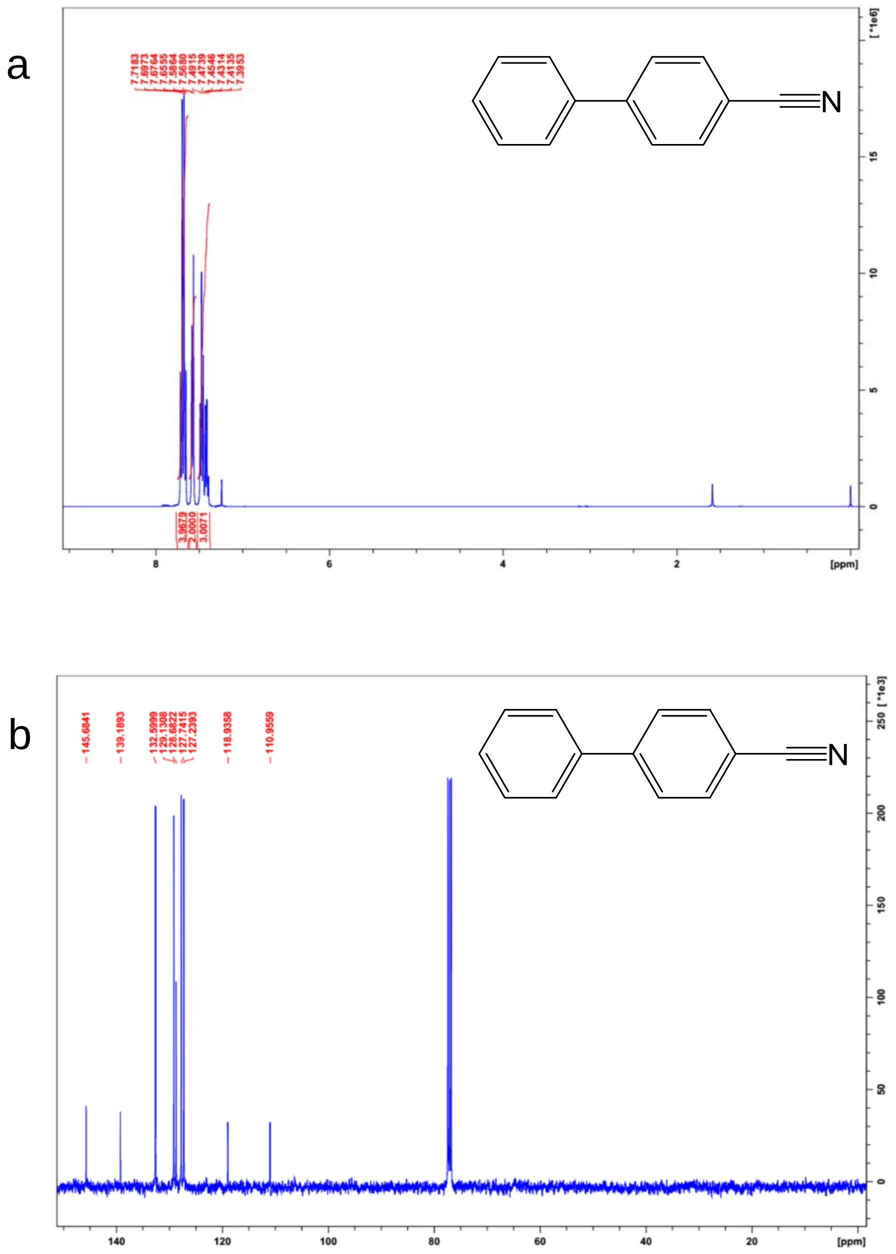

Supplementary Figure 41 a. ^{1}H NMR and b. ^{13}C NMR spectra.

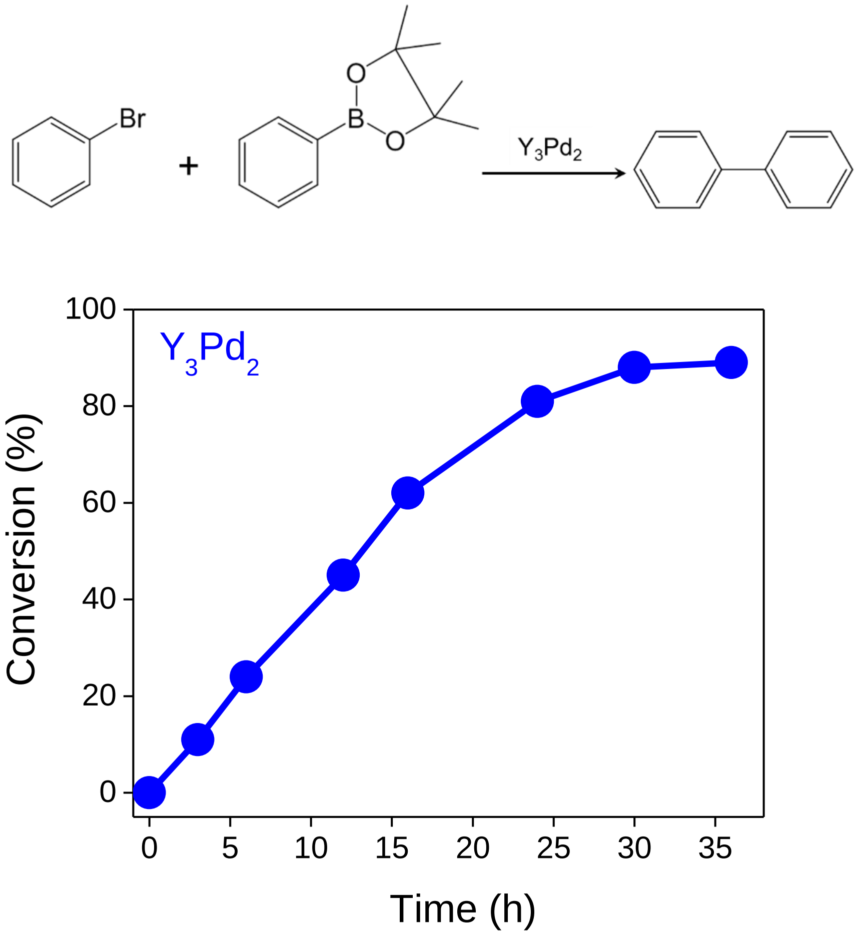

Supplementary Figure 42 Time-dependent catalysis over Y_3Pd_2 catalyst for Suzuki coupling reaction of bromobenzene with phenyl boronic acid pinacol ester. Reaction conditions: Pd (40 mol% relative to organohalide); 0.5 mmol bromobenzene, 0.8 mmol phenylboronic acid pinacol ester, 1.5 mmol K_2CO_3, 5 mL solvent, 60 °C. An error analysis was performed for these data and the error range was determined to be ±5%.

Supplementary Table 1 Catalytic performance of Y_3Pd_2 and Pd

Sample	iodobenzene			bromobenzene		
	TOFs based on surface Pd by unit cell (h^{-1})	TOFs based on total Pd (h^{-1})	Carbon balance	TOFs based on surface Pd by unit cell Pd (h^{-1})	TOFs based on total Pd (h^{-1})	Carbon balance
Y_3Pd_2	1670.1	7.6	98.1%	4034.4	7.33	99.1%
Pd	129.3	0.9	98.4%	355	2.25	98.9%

Reaction conditions: Pd (40 mol% relative to organohalide); 0.5 mmol organohalide, 0.8 mmol phenylboronic acid, 1.5 mmol K_2CO_3, 5mL solvent, iodides 30 °C, bromides 60 °C. Carbon balance was calculated by the consumption of the substrate iodobenzene and the yield of the biphenyl, respectively, through the analysis of GC and GC-MS. An error analysis was performed for these data and the error range was determined to be ±5%.

Supplementary Table 2 Ullmann coupling reactions of iodobenzene with iodobenzene over Y_3Pd_2 and pure Pd.

Entry	Catalyst	Substrate	Product	Time (h)	Yield (%)
1	Y_3Pd_2	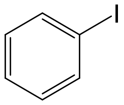	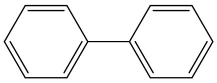	24	trace
2	Pd	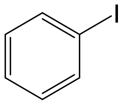	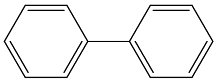	24	trace

Reaction conditions: Pd (40 mol% relative to organohalide); 0.5 mmol iodobenzene, 1.5 mmol K_2CO_3, 5 mL solvent, 30 °C. An error analysis was performed for these data and the error range was determined to be ±5%.

Supplementary Table 3 Catalytic performance comparison of Y_3Pd_2 with commercial heterogeneous Pd-based catalysts.

Catalyst	Surface area ($m^2\ g^{-1}$)	CO $_{ads.}$ ($ml\ g^{-1}$)	Exposed Pd by CO $_{ads.}$ ($10^{-6} mol\ g^{-1}$)	Reaction rate ($mmol\ g^{-1}h^{-1}$)	TOFs (h^{-1})
[a] Y_3Pd_2	0.431	0.0198	0.9	3.04	3378
[b] Y_3Pd_2	3.2	0.1312	5.9	17.02	2934
[c] Pd/C	526.5	0.8810	78.6	15.2	193
[d] Pd/Al_2O_3	96.8	1.991	177.8	14.1	79
[e] $Pd\text{-}Pb/CaCO_3$	6.9	0.1496	13.4	5.98	446

Reaction conditions: [a]Pd (40 mol% relative to organohalide); [b]Y_3Pd_2 ball-milled by ethanol, Pd (40 mol% relative to organohalide); [c]10wt%Pd/C (20 mg); [d]5wt%Pd/Al_2O_3 (20 mg); [e]5wt%Pd Lindlar catalyst (20 mg); 0.5 mmol iodobenzene, 0.8 mmol phenylboronic acid, 1.5 mmol K_2CO_3, 5mL solvent, 30 °C. A stoichiometry of Pd/CO = 1 was used for Y_3Pd_2 and Pd/CO = 2 for Pd/C, Pd/Al_2O_3 and Lindlar catalyst. An error analysis was performed for these data and the error range was determined to be ±5%.

Supplementary Table 4 Summary of results reported for different Pd-based catalysts in the coupling of iodobenzene and phenylboronic acid

Catalyst	Condition	TOF (h^{-1})	Recycle No	Reference
**Y_3Pd_2**	**EtOH, K_2CO_3 30 °C, 1 h**	**1670.1**	**20**	**This work**
Pd/MFC	EtOH, K_2CO_3 Reflux, 1 h	324.6	5	2
Pd NPs	H_2O, Na_2CO_3 80 °C, 24h	40.5	6	3
Pd/Fe_3O_4	DME-H_2O, Na_2CO_3 Reflux, 24h	41.3	10	4
Pd@Mag-MSN	CH_2Cl_2, K_2CO_3 80 °C, 6h	14.2	3	5
Pd/CNT-SiC	EtOH-H_2O, K_3PO_4 80 °C, 1h	350	6	6
Pd@CNPCs	DMF-H_2O, K_2CO_3 50 °C, 1.5h	644.6	5	7
MUA-Pd	DMF, NaOH 90 °C, 8h	6.2	5	8
Pd-CNT-ED-OH	DMF, Na_2CO_3 110 °C, 24h	13.1	7	9
Pd(0)/MCoS-1	H_2O, K_2CO_3 70 °C, 5h	98	6	10
Pd/$NiFe_2O_4$	DMF-H_2O, Na_2CO_3 90 °C, 5mins	196	4	11
Sugar-derived PdNPs	iPrOH,KCO_3 100 °C, 20h	4.4	4	12
G/MWCNTs/Pd	EtOH-H_2O, K_2CO_3 60 °C, 15mins	799	6	13

Supplementary Table 5 Comparison of the activity of Y_3Pd_2 with other results reported Pd-based heterogeneous photocatalyzed Suzuki reactions under visible light

Catalyst	Condition	TOF (h^{-1})	Recycle No	Reference
**Y_3Pd_2**	**EtOH, K_2CO_3 30 °C, 1 h**	**1670.1** (iodobenzene)	**20**	**This work**
Pd/SiC	DMF-H_2O, Cs_2CO_3 30 °C, 80mins	1053 (iodobenzene)	5	14
m-CNR-Pd	EtOH-H_2O, K_2CO_3 RT, 1h	51.6 (iodobenzene)	6	15
Pd/Au/PN-CeO_2	DMF-H_2O, K_2CO_3 30 °C, 30mins	6.3 (iodobenzene)	6	16
**Y_3Pd_2**	**EtOH, K_2CO_3 60 °C, 30mins**	**4034.4** (bromobenzene)	---	**This work**
Au-Pd alloy NP	DMF-H_2O, K_2CO_3 30 °C, 3h	14.5 (bromobenzene)	---	17
Au-Pd Nanostructure	H_2O, NaOH 25 °C, 1h	162 (bromobenzene)	---	18
**Y_3Pd_2**	**EtOH, K_2CO_3 30 °C, 1h**	**1432.6** (iodotoluene)	---	**This work**
PDA/Pd NPs	DMF-H_2O, K_2CO_3 RT, 2h	85 (iodotoluene)	---	19
WS_2/Pd NPs	EtOH-H_2O, K_2CO_3 RT , 3 h	1244 (iodotoluene)	---	20

Supplementary Table 6 Suzuki cross-coupling reactions of chlorobenzene with phenylboronic acid over Y_3Pd_2.

Entry	Aryl Halides	Arylboronic Acids	Product	Time (h)	Yield (%)
1	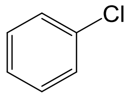	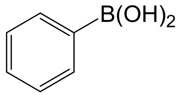	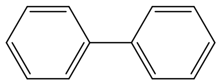	24	7

Reaction conditions: Pd (40 mol% relative to organohalide); 0.5 mmol chlorobenzene, 0.8 mmol phenylboronic acid, 1.5 mmol K_2CO_3, 5 mL solvent, 60 °C. An error analysis was performed for these data and the error range was determined to be ±5%.

Supplementary Table 7 Characterization and activity of fresh and ball-milled Y_3Pd_2.

Sample	S_{BET} (m^2g^{-1})	CO $_{ads.}$ (ml g^{-1})	Exposed Pd by CO $_{ads.}$ (10^{-6}mol g^{-1})	Exposed Pd by unit cell (10^{-6}mol g^{-1})	Reaction rate (mmol $g^{-1}h^{-1}$)	TOFs by CO $_{ads.}$ (h^{-1})	TOFs by unit cell. (h^{-1})
$^{a}Y_3Pd_2$	0.43	0.0198	0.9	1.8	3.04	3378	1670
$^{a}Y_3Pd_2$ BM by heptane	1.1	0.0617	2.7	4.6	6.41	2374	1393
$^{a}Y_3Pd_2$ BM by ethanol	3.2	0.1312	5.9	13.4	17.02	2934	1270
$^{b}Y_3Pd_2$	0.43	0.0198	0.9	1.8	0.37	411	204

[a] Reaction conditions: Pd (40 mol% relative to organohalide); 0.5 mmol iodobenzene, 0.8 mmol phenylboronic acid, 1.5 mmol K_2CO_3, 5mL solvent, 30 °C. [b] Pd (40 mol% relative to organohalide); 0.5 mmol bromobenzene, 0.8 mmol phenylboronic acid pinacol ester, 1.5 mmol K_2CO_3, 5 mL solvent, 60 °C. An error analysis was performed for these data and the error range was determined to be ±5%.

Supplementary Table 8 Bader charges of atoms on Y_3Pd_2(0001) surface before and after C_6H_5Br adsorption.

	Pd	Y1	Y2	Y3	C_6H_5Br
Y_3Pd_2 bulk	–1.88	+1.39	+1.19	+1.23	
Y_3Pd_2(0001) surface	–1.77	+1.51	+1.49	+1.28	
C_6H_5Br@Y_3Pd_2(0001) surface	–1.69	+1.51	+1.51	+1.28	–0.12

The Bader charges of atoms in Y_3Pd_2 bulk and The Bader charge of the C_6H_5Br molecule as a whole is also given. The results indicate a charge transfer of 0.12e^- from the Y_3Pd_2(0001) surface (mainly from the active Pd site) to the C_6H_5Br molecule.

Supplementary Table 9 Catalytic performance of Y_3Pd_2, Y_3Pd, Y_3Pd_4, YPd_3, Pd, Y_5Si_3 and Y powder.

Catalyst	Surface area ($m^2\ g^{-1}$)	Exposed Pd by unit cell (10^{-8}mol)	Reaction rate (mmol $g^{-1}h^{-1}$)	Reaction rate (mmol $m^{-2}h^{-1}$)	TOFs (h^{-1})
[a] Y_3Pd_2	0.431	9.1	3.04	7.05	1670
[a] YPd_3	0.081	1.2	0.63	7.84	1562
[a] Y_3Pd_4	0.143	3.5	1.23	7.04	1291
[a] Y_3Pd	0.287	4.2	0.85	3.01	1578
[a] Pd	0.87	14	0.83	2.16	129
[b] Y_5Si_3	1.0	--	N.D.	N.D.	---
[c] Y powder	0.21	--	N.D.	N.D.	---

Reaction conditions: [a] Pd (40 mol% relative to organohalide); [b]Y_5Si_3 (50 mg); [c]Y powder (50 mg); 0.5 mmol iodobenzene, 0.8 mmol phenylboronic acid, 1.5 mmol K_2CO_3, 5mL solvent, 30 °C. An error analysis was performed for these data and the error range was determined to be ±5%.

Supplementary References

1. Park, I. et al. Atomic layer deposition of Y_2O_3 films using heteroleptic liquid $(iPrCp)_2Y(iPr\text{-}amd)$ precursor, *J. Mater. Chem. C* **2**, 9240−9247 (2014).
2. Zhu, M. & Diao, G. Magnetically recyclable Pd nanoparticles immobilized on magnetic Fe_3O_4@C nanocomposites: Preparation, characterization, and their catalytic activity toward suzuki and heck coupling reactions. *J. Phys. Chem. C* **115**, 24743−24749 (2011).
3. Zhang, A. et al. Homogeneous Pd nanoparticles produced in direct reactions: green synthesis, formation mechanism and catalysis properties. *J. Mater. Chem. A* **2**, 1369−1374 (2014).
4. Jiang, Y. et al. Simple synthesis of Pd–Fe_3O_4 heterodimer nanocrystals and their application as a magnetically recyclable catalyst for Suzuki cross-coupling reactions. *Phys. Chem. Chem. Phys.* **13**, 2512−2516 (2011).
5. Shylesh, S., Wang, L., Demeshko, S. & Thiel, W. R. Facile synthesis of mesoporous magnetic nanocomposites and their catalytic application in carbon–carbon coupling reactions. *ChemCatChem* **2**, 1543−1547 (2010).
6. Yuan, H., Liu, H., Zhang, B., Zhang, L., Wang, H. & Su, D. S. A Pd/CNT-SiC monolith as a robust catalyst for Suzuki coupling reactions. *Phys. Chem. Chem. Phys.* **16**, 11178−11181 (2014).
7. Zhang, P., Weng, Z., Guo, J. & Wang, C. Solution-dispersible, colloidal, conjugated porous polymer networks with entrapped palladium nanocrystals for heterogeneous catalysis of the Suzuki–miyaura coupling reaction. *Chem. Mater.* **23**, 5243−5249 (2011).
8. Cargnello, M. et al. A versatile approach to the synthesis of functionalized thiol-Protected palladium nanoparticles. *Chem. Mater.* **23**, 3961−3969 (2011).
9. Kim, E., Jeong, H. S. & Kim, B. M. Studies on the functionalization of MWNTs and their application as a recyclable catalyst for C-C bond coupling reactions. *Catal. Commun.* **46**, 71−74 (2014).
10. Roy, A. S. et al. Pd-grafted porous metal–organic framework material as an efficient and reusable heterogeneous catalyst for C–C coupling reactions in water. *Appl. Catal. A* **469**, 320−327 (2014).
11. Borhade, S. R. & Waghmode, S. B. Studies on Pd/$NiFe_2O_4$ catalyzed ligand-free Suzuki reaction in aqueous phase: synthesis of biaryls, terphenyls and polyaryls. *Beilstein J. Org. Chem.* **7**, 310−319 (2011).
12. Camp, J. E. et al. Glucose-derived palladium(0) nanoparticles as in situ-formed catalysts for Suzuki–miyaura cross-coupling reactions in isopropanol. *ACS Sustainable Chem. Eng.* **2**, 500−505 (2014).

13. Song, H. Q., Zhu, Q., Zheng, X. & Chen, X. G. One-step synthesis of three-dimensional graphene/multiwalled carbon nanotubes/Pd composite hydrogels: an efficient recyclable catalyst for Suzuki coupling reactions. *J. Mater. Chem. A* **3**, 10368−10377 (2015).
14. Jiao, Z., Zhai, Z., Guo, X. & Guo, X. Y. Visible-light-driven photocatalytic Suzuki–miyaura coupling reaction on mott–schottky-type Pd/SiC catalyst. *J. Phys. Chem. C* **119**, 3238−3243 (2015).
15. Li, X. H., Baar, M., Blechert, S. & Antonietti, M. Facilitating room-temperature Suzuki coupling reaction with light: Mott-Schottky photocatalyst for C-C-coupling. *Sci. Rep.* **3**, 1743 (2013).
16. Zhang, S. et al. Visible-light-activated Suzuki–miyaura coupling reactions of aryl chlorides over the multifunctional Pd/Au/porous nanorods of CeO_2 catalysts. *ACS. Catal.* **5**, 6481−6488 (2015).
17. Xiao, Q. et al. Efficient photocatalytic Suzuki cross-coupling reactions on Au–Pd alloy nanoparticles under visible light irradiation. *Green Chem.* **16**, 4272−4285 (2014).
18. Wang, F. et al. Plasmonic harvesting of light energy for Suzuki coupling reactions. *J. Am. Chem. Soc.* **135**, 5588−5601 (2013).
19. Xie, A. et al. Polydopamine nanofilms as visible light-harvesting interfaces for palladium nanocrystal catalyzed coupling reactions. *Catal. Sci. Technol.* **6**, 1764−1771 (2016).
20. Raza, F., Yim, D., Park, J. H., Kim, H. I., Jeon, S. J. & Kim, J. H. Structuring Pd nanoparticles on 2H-WS_2 nanosheets induces excellent photocatalytic activity for cross-coupling reactions under visible light. *J. Am. Chem. Soc.* **139**, 14767−14774 (2017).
